# Revision of the perceived parental-educational-anxiety scale and its application among Chinese college freshmen

**DOI:** 10.1371/journal.pone.0358097

**Published:** 2026-09-16

**Authors:** Laiju Du, Zongpei Dai, Xiaoxiao Sun, Ming Liu, Qin Dai

**Affiliations:** 1 Department of Pain, Southwest Hospital, Army Medical University, Chongqing, China; 2 Research center for Children’s Brain and Cognitive Sciences, Chongqing University of Education, Chongqing, China; University of Louisiana at Lafayette, UNITED STATES OF AMERICA

## Abstract

**Background:**

Parental educational anxiety is prevalent globally and impacts children’s psychological outcomes profoundly. However, children’s perceived parental educational anxiety (PPEA) remains understudied, and a standardized tool for PPEA is lacking. Furthermore, the level and influencing factors of PPEA among children remain unclear. This study aimed to revise the Parental Educational Anxiety Scale from a parental perspective to a child-perceived perspective and to further explore its level and influencing factors in college freshmen.

**Methods:**

The Perceived Parental Educational Anxiety Scale (PPEAS) was revised via pronoun conversion of the original items and expert consultation. Two independent samples were recruited for scale validation: Sample 1 (n = 232) for exploratory factor analysis (EFA), and Sample 2 (n = 287) for confirmatory factor analysis (CFA) and reliability testing. Subsequently, 646 participants completed the revised PPEAS, the Family Environment Scale-Chinese Version (FES-CV), and the Connor-Davidson Resilience Scale (CD-RISC). Non-parametric tests and hierarchical multiple linear regressions were performed.

**Results:**

EFA identified a three-factor structure consisting of 16 items with adequate model fit (χ²/df = 2.691, RMSEA = 0.077, TLI = 0.919, CFI = 0.932). Analyses confirmed satisfactory internal consistency reliability (Cronbach’s α = 0.922) and test-retest reliability (ICC = 0.825). The median PPEA score of Chinese college freshmen was 40 (IQR: 32, 49). Hierarchical regression showed that family conflict was positively associated with PPEA, while children’s resilience was negatively associated with PPEA. Contrary to our hypothesis, family cohesion was not a significant predictor in the final model.

**Conclusion:**

The revised PPEAS exhibits good reliability and validity, providing a reliable instrument for measuring perceived educational anxiety from the children’s perspective. The findings highlight that reducing family conflict and improving children’s resilience may contribute to lower PPEA levels.

## 1. Introduction

In recent years, a growing body of research has examined education-related anxiety, including students’ academic anxiety and parental educational anxiety [1,2]. The latter has become a notable concern worldwide, particularly in East Asian contexts such as China [3]. Parental educational anxiety refers to a persistent emotional state of urgency, tension, and worry experienced by parents due to uncertainties regarding their children’s education [4]. This anxiety is prevalent in China due to the intense “involutionary” competition in Chinese society, especially within the education sector [5,6]. In response, the Chinese government issued the “Double Reduction” policy in July 2021, which aims to reduce students’ academic burden and alleviate parental educational anxiety [7]. Although certain progress has been made over the past few years, existing studies indicate that educational anxiety remains widespread among parents [8,9].

*Family system theory* posits that the family serves as an interactive system in which members’ emotions influence each other [10]. In this system, children actively perceive and interpret parental behaviors and emotions, which determines their own psychological developments. However, there are often discrepancies between parental self-reports and children’s perceptions regarding parental behaviors and emotions [11–13]. As revealed by existing research, children’s perceptions of parental general negative emotions (e.g., depression) are generally lower than parental self-reports [14,15], likely because children have limited access to their parents’ internal affective states [15,16]. Similarly, children’s perceived parental educational anxiety may diverge from parental self-reports. Notably, compared to parents’ self-reports, children’s perceptions can be more effective in predicting children’s mental health. This view is supported by cognitive theories of emotion. It is proposed that an individual’s interpretation outweighs the objective event in shaping emotional response, and by empirical evidence. For instance, adolescents’ perceived parental control predicts their depression more strongly than parents’ self-reporting [12,17]. Thus, using parental self-report instruments to assess children’s PPEA is unreasonable, highlighting the necessity of a reliable scale specifically designed for children. Nevertheless, the existing instruments such as the questionnaire of parents’ anxiety about their children’s education (MQPAE) [18], the self-report parental anxiety over children’s education scale (PACES) [19], and the parental educational anxiety scale (PEAS) [20], are all designed for parental self-reports. Among the above-mentioned tools, the PEAS developed by Yiwen Tang is a widely utilized, valid, and reliable measurement scale for parents of primary and secondary school students [20]. However, to date, no tool is available to assess children’s PPEA. Thus, the level of PPEA, which can be employed to identify high-risk individuals, remains largely unknown. To address this problem, the present study aims to develop a dedicated PPEA scale and focuses on Chinese college freshmen as the target population for two reasons. First, given that they have just completed 12-year basic education in China, their retrospective reports reflect an integrated and long-term perception of parental educational anxiety. Additionally, college students are prone to various mental disorders, with a depression prevalence of 33.6% and an anxiety prevalence of 39.0%, respectively [21]. Drawing on evidence linking perceived parental negative emotions to poor mental health in children, PPEA may exert a similar adverse impact, underscoring the urgent need for a screening tool to identify individuals with high PPEA levels.

Moreover, PPEA may be influenced by several factors. Based on ecological systems theory [22], the family constitutes the earliest and most fundamental micro-system for children’s development [23]. At the *family level*, family cohesion and family conflict may play a role in anxiety transmission. Specifically, a family climate characterized by high conflict and low cohesion may amplify children’s PPEA by undermining their emotional security and heightening vigilance toward negative emotional cues [24]. Conversely, a cohesive family environment can buffer this perception by serving as a secure base and providing shared coping resources [25]. Furthermore, specific interaction patterns (e.g., parenting styles) are of great significance to the transmission of educational anxiety [26,27]. For example, an authoritarian parenting style may increase children’s PPEA by enhancing their sensitivity to parental negative emotions [28]. At the *individual* level, resilience serves as a psychological resource that determines individuals’ capacity to cope with such anxiety signals [29,30]. Specifically, resilient children are more likely to employ adaptive strategies such as cognitive reappraisal, which allows them to interpret parental anxiety in less threatening terms, thereby reducing their perceived anxiety [31]. Thus, it could be assumed that family cohesion, family conflict, and children’s resilience may influence the formation of PPEA.

In brief, this study aimed to revise the Parental Educational Anxiety Scale from the parental perspective to the children’s perspective, and further examine the level and influencing factors of PPEA among Chinese college freshmen. It was hypothesized that family conflict would be positively associated with PPEA, whereas family cohesion and children’s resilience would be negatively associated with PPEA.

## 2. Materials and methods

### 2.1. Participants

College freshmen from two universities in Chongqing, China, were recruited for this survey from April to July 2025. Convenience sampling was adopted to enroll participants through advertising from different departments. The inclusion criteria were as follows: first-year undergraduate students, voluntary participation, and signing on written informed consent. Exclusion criteria from self-reported questions included: current or history of mental disorders (major depression, bipolar disorder, anxiety disorder, et al), substance or drug abuse, or severe physical disease.

Three independent samples were recruited. **Sample 1** (n = 232; 31 females; mean age = 18.24 years) was utilized for item analysis and exploratory factor analysis (EFA). **Sample 2** (n = 287; 177 females; mean age = 18.02 years) was used for confirmatory factor analysis (CFA), as well as the assessments of criterion-related validity, convergent validity, discriminant validity, and internal consistency reliability. **Sample 3** (n = 646; 231 females; mean age = 19.13 years) was applied to observe the level and influencing factors of PPEA.

### 2.2. Scale revision

#### Original scale used for revision.

The revision was carried out based on the 16-item PEAS developed by Yiwen Tang in 2025 [20]. The PEAS can measure parental educational anxiety by academic performance, competitive environment, and educational efficacy. The respective item scores on a 5-point Likert scale that ranges from 1 (*Never*) to 5 (*Always*). Total score ranges from 16 to 80, with higher score representing stronger educational anxiety. The scale exhibits good reliability and validity among parents of primary and secondary school students [20].

First, items were converted from self-reporting style to children’s perspective (e.g., “*I feel anxious*” was converted to “*My parents feel anxious*”). To be specific, the assessment of parents’ internal experiences shifts to the assessment of children’s perceptions of these experiences. Next, the content validity of the adapted items was evaluated by 10 experts (one professor and nine doctoral candidates with professional experience in educational or psychology). These experts rated the relevance of each item independently to the intended construct on a 4-point Likert scale (1 = not relevant, 4 = highly relevant). The item-level Content Validity Index (I-CVI) values for the 16 items ranged from 0.90 to 1.00 (> 0.78). Moreover, the scale-level CVI (S-CVI) based on the universal agreement method reached 0.88 (>0.80). The above-mentioned results indicated good content validity of the draft scale. Finally, given that the revised scale was designed for college freshmen, a population in emerging adulthood, 5 college freshmen were recruited for semi-structured interviews to examine face validity and comprehensibility. The interview guide focused on participants’ comprehension of each item of PPEAS, as well as any supplement for perceived parental-educational-anxiety, identification of any ambiguous wording or new suggestion, and confirmation that the scale and items clearly measured perceived parental educational anxiety from a child-perceived perspective. Based on the feedback, item 15 was revised from “My parents were uncertain whether their current parenting approach was the best for me” to “My parents were uncertain whether their parenting approach was the best for me” to match the retrospective design. No other major modifications were conducted. The final 16-item perceived parental educational anxiety scale (PPEAS) was in S1 Table. Subsequently, sample 1 (n = 232) and sample 2 (n = 287) were employed to examine the reliability and validity of the scale. Six weeks later, 156 participants from sample 2 completed the questionnaire again to assess test-retest reliability.

Item analysis was examined item-total correlations (items with *r* > 0.40 were retained) [32] and comparing extreme groups (the top and bottom 27% of scores) via the Mann-Whitney U test. EFA was subsequently conducted using Principal Axis Factoring (PAF) with promax oblique rotation. The criterion was the Kaiser-Meyer-Olkin (KMO) value > 0.60 and Bartlett’s sphericity test (*p* < 0.05) [33,34]. Factors with eigenvalues > 1 were retained. Individual items were retained based on the following thresholds: factor loadings > 0.50, communality > 0.30, and cross-loading differences > 0.20 [35]. A cumulative explained variance of 40–60% was considered as adequate [36]. Subsequently, CFA was performed. Model fit was considered acceptable when χ²/df < 3, RMSEA < 0.08, TLI > 0.90, and CFI > 0.90 [37–39]. Convergent validity was assessed using composite reliability (CR > 0.60) and average variance extracted (AVE > 0.50) [40]. The heterotrait-monotrait ratio (HTMT < 0.85) was used to assess discriminant validity [41]. Additionally, criterion-related validity was examined by Spearman correlation with generalized anxiety and parental parenting styles. Reliability was verified using Cronbach’s α coefficient (> 0.7) for internal consistency reliability and intra-class correlation coefficient (ICC > 0.60) [42,43]. Lastly, measurement invariance was tested across gender groups through multi-group confirmatory factor analysis.

### 2.3. Study instruments

#### Self-designed Questionnaire.

A self-designed questionnaire was designed based on a review of the literature [7,44], covering demographic variables including gender, age, only-child status, parental marital status, parental education level, and family economic status. Consistent with previous studies [45,46], two variables were measured using single-item measures: parenting style (*Authoritarian*, *Permissive*, *Indulgent*, *Democratic*) and primary academic supervisor (*Neither*, *Father*, *Mother*, *Both*).

#### The Family Environment Scale-Chinese Version (FES-CV).

This scale was originally developed by Moos et al. [47], and its Chinese version was revised by Lipeng Fei [48]. The scale consists of 90 true-or-false items divided into 10 subscales, each comprising 9 items. The present study adopted the Cohesion and Conflict subscales, whose Cronbach’s α coefficients were 0.795 and 0.702, respectively.

#### The Chinese version of the Connor-Davidson Resilience Scale (CD-RISC).

The original CD-RISC was compiled by Connor and Davidson, and its Chinese version, revised by Yun Xu for Chinese student populations, contains 22 items [32].This scale adopts a 5-point Likert scoring system (1 = “*Completely disagree*”, 5 = “*Completely agree*”),with total scores ranging from 22 to 110. Higher total scores indicate greater resilience. In this study, the Cronbach’s α coefficient of the scale was 0.963.

#### Criterion instruments.

According to emotional contagion theory [49], PPEA is positively associated with children’s anxiety. Given that parental educational anxiety is frequently manifested through controlling and less warm parenting practices, PPEA is likely positively correlated with parental rejection and overprotection, and negatively correlated with parental emotional warmth [7,50].

#### The Chinese version of the 7-item Generalized Anxiety Disorder (GAD-7).

The GAD-7 was developed by Spitzer et al. [51], and its Chinese version has been validated among Chinese university students by Zhang et al. [52]. This 7-item scale adopts a 4-point Likert scale (0 = “*Not at all*”, 3 = “*Nearly every day*”), with total scores ranging from 0 to 21; higher scores indicate higher levels of anxiety [51]. The Cronbach’s α coefficient of the scale in the current study was 0.842.

#### The Chinese version of the shortened Egna Minnen Beträffande Uppfostran (S-EMBU-C).

The Egna Minnen Beträffande Uppfostran (EMBU) was originally developed by Perris et al. [53]. The Chinese short-form version, consisting of 42 items, was revised by Jiang Jiang [54]. It contains paternal and maternal subscales, each covering three dimensions: rejection, emotional warmth, and overprotection. A 4-point Likert scale is applied (1 = “*Never*”, 4 = “*Always*”). A higher total score for each dimension indicates a more prominent corresponding parenting style. In this study, the Cronbach’s α coefficients ranged from 0.746 to 0.911 (paternal: rejection = 0.783, overprotection = 0.746, warmth = 0.903; maternal: rejection = 0.864, overprotection = 0.799, warmth = 0.911).

### 2.4. Procedure

This study was approved by the Ethics Committee of the Army Medical University (Approval No. 2024-31-02). Participants were recruited from two universities in Chongqing. Prior to the survey, the purpose, voluntary nature and confidentiality were clearly explained to potential participants, and written informed consent was obtained. Subsequently, the questionnaires were distributed by two trained psychological professionals in papers. Furthermore, questionnaires with incomplete or patterned responses were excluded from analysis. Participants could withdraw at any time without any negative consequences. They received a gift after completing the questionnaires.

### 2.5. Statistical analysis

Statistical analyses were conducted through SPSS 26.0, AMOS 26.0 and R 4.5.1. PPEA scores were described using the median and interquartile range due to non-normal distribution. Non-parametric tests were carried out for univariate analyses. Specifically, the Mann-Whitney U test was performed for two-group comparisons, Kruskal-Wallis H test was performed for multi-group comparisons, and Spearman correlation was used for continuous variables. Diagnostic statistics were examined in accordance with standard regression diagnostic procedures, with the aim of detecting potential influential observations (e.g., high leverage points) [55]. Subsequently, hierarchical regressions were conducted to observe predictors of PPEA levels: Model 1 included socio-demographic variables, Model 2 added family environmental variables, and Model 3 further incorporated individual resilience. All categorical variables were dummy-coded (Table 1).

**Table 1:** Dummy variable coding of categorical variables.

Categorical variables	Dummy variable coding
Gender	Female = 0; Male = 1
Parental marital status	Not divorced = 0; Divorced = 1
Paternal education level	University (Z1 = 0, Z2 = 0); Senior high school (Z1 = 1, Z2 = 0); Junior high school or below (Z1 = 0, Z2 = 1)
Maternal education level	University (Z1 = 0, Z2 = 0); Senior high school (Z1 = 1, Z2 = 0); Junior high school or below (Z1 = 0, Z2 = 1)
Family economic status	Poor (Z1 = 0, Z2 = 0); Average (Z1 = 1, Z2 = 0); Well-off (Z1 = 0, Z2 = 1)
Parental parenting styles	Democratic (Z1 = 0, Z2 = 0, Z3 = 0); Authoritarian (Z1 = 1, Z2 = 0, Z3 = 0); Permissive (Z1 = 0, Z2 = 1, Z3 = 0); Indulgent (Z1 = 0, Z2 = 0, Z3 = 1)
Primary academic supervisor	Neither parent (Z1 = 0, Z2 = 0, Z3 = 0); Father (Z1 = 1, Z2 = 0, Z3 = 0); Mother (Z1 = 0, Z2 = 1, Z3 = 0); Both parents (Z1 = 0, Z2 = 0, Z3 = 1)

Harman’s single-factor test was performed to evaluate potential common method bias. The first unrotated factor explained27.709% of the total variance, which was lower than50% [56], indicating no obvious common method bias in this study.

## 3. Results

### 3.1. Reliability and validity tests of the scale

#### 3.1.1. Item analysis.

Item-total correlations were calculated between each item and the total score of the full scale (**Table 2**). All items exhibited significant correlations with the total score, ranging from 0.418 to 0.725. In addition, significant differences in all item scores were observed between the high and low groups (*Z* = 5.955–9.426, *p* < 0.001). All 16 items satisfied the retention criteria and were retained for subsequent analyses.

**Table 2:** Item analysis and exploratory factor analysis(n = 232).

Items	Item-total correlations				Z value	Factor loading			Communality
	Academic performance	Competitive environment	Educational efficacy	Full scale		Academic performance	Competitive environment	Educational efficacy	
A1	0.568			0.470	6.217***	0.661			0.329
A2	0.720			0.712	8.997***	0.584			0.578
A3	0.759			0.725	8.892***	0.622			0.609
A4	0.740			0.670	9.062***	0.877			0.631
A5	0.692			0.638	8.516***	0.698			0.502
A6	0.680			0.673	8.323***	0.616			0.562
A7	0.726			0.719	9.019***	0.612			0.730
A8	0.755			0.716	8.600***	0.905			0.694
A9	0.552			0.529	7.635***	0.512			0.330
B1		0.667		0.713	9.426***		0.529		0.588
B2		0.707		0.599	7.872***		0.618		0.488
B3		0.645		0.581	8.056***		0.854		0.615
B4		0.664		0.589	8.062***		0.668		0.525
C1			0.494	0.429	5.955***			0.510	0.377
C2			0.646	0.469	6.608***			0.745	0.649
C3			0.583	0.418	6.495***			0.669	0.501

#### 3.1.2. Exploratory factor analysis.

The results confirmed the suitability of the data for factor analysis (KMO = 0.913, Bartlett’s sphericity test *p* < 0.001). EFA on sample1 extracted three factors with eigenvalues greater than 1, which was consistent with the structure of the original scale. All 16 items yielded factor loadings above 0.50 on their respective dominant factors, with cross-loading differences exceeding 0.20 (**Table 2**). The three factors collectively explained 62.740% of the cumulative variance.

#### 3.1.3. Confirmatory Factor Analysis.

**Fig 1:**
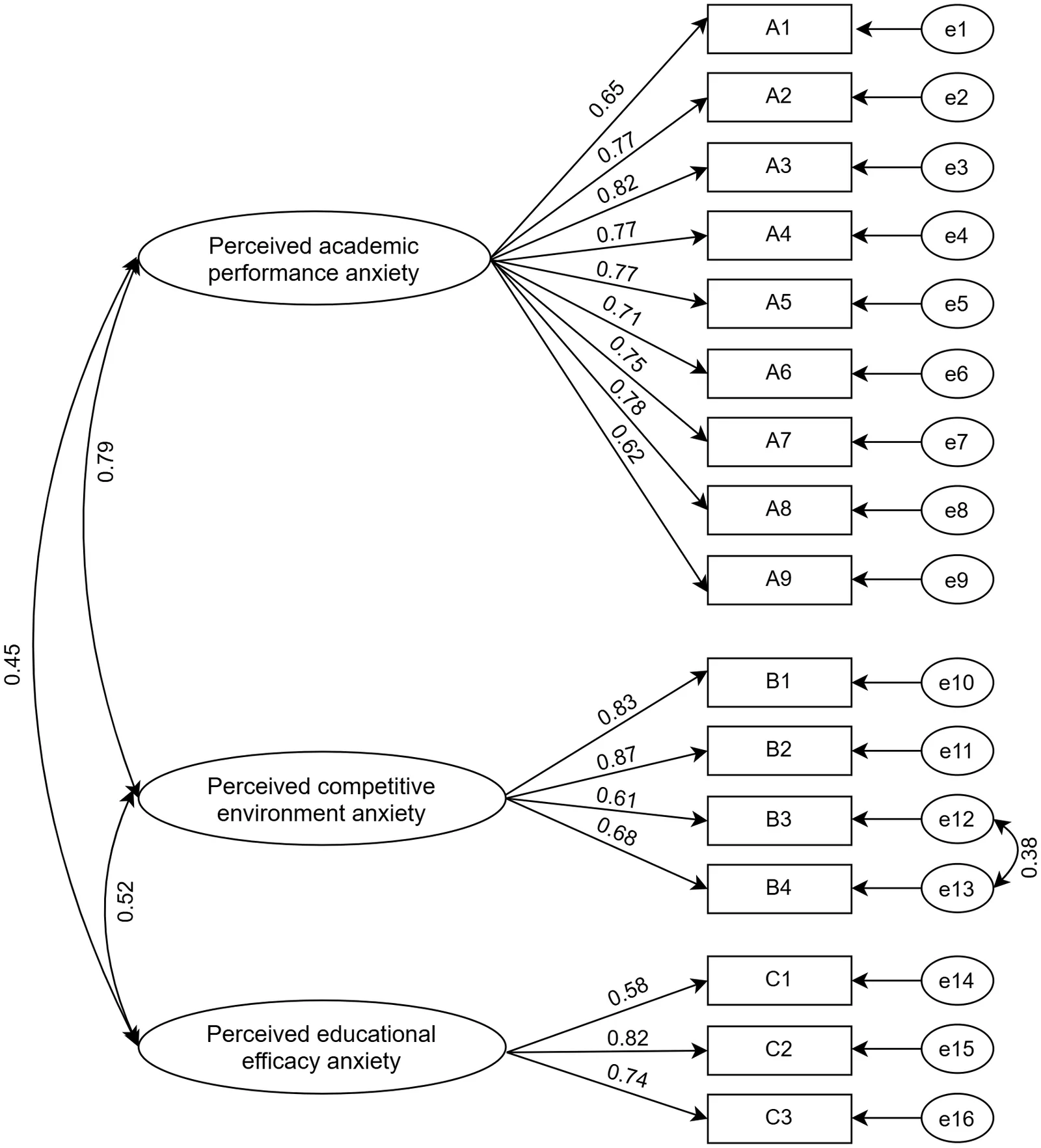
Confirmatory factor analysis for three-dimensional PPEAS.

CFA was performed (Fig 1). The initial model fit was suboptimal (χ²/df = 3.032, RMSEA = 0.084, TLI = 0.902, CFI = 0.918). When the modification indices (MI) exceed 10 and the items share similar content and wording, residual correlation can be added between them [57]. Items 12 and 13 belong to same dimension and possess similar wording, with MI exceeding the recommended threshold, providing a reasonable theoretical basis for correlated measurement errors. Therefore, error covariance was conducted between Items 12 and 13.As this modification was limited to a single pair of the items and was theoretically grounded, the construct validity of the scale is unlikely to be compromised. After modification, the model fit was improved (χ²/df = 2.691, RMSEA = 0.077, TLI = 0.919, CFI = 0.932) and met standard psychometric criteria.

#### 3.1.4. Convergent validity and discriminant validity.

The AVE values ranged from 0.520 to 0.592, the CR values ranged from 0.629 to 0.853, and the HTMT values ranged from 0.496 to 0.770 (**Table 3**).

**Table 3:** Convergent validity and discriminant validity (n = 287).

	AVE	CR	Academic performance	Competitive environment	Educational efficacy
Academic performance	0.543	0.853	1.000		
Competitive environment	0.592	0.774	0.770	1.000	
Educational efficacy	0.520	0.629	0.496	0.547	1.000

#### 3.1.5. Criterion-related validity.

Higher total scores of PPEA were significantly and positively correlated with paternal and maternal parental rejection (paternal: *r* = 0.438; maternal: *r* = 0.400), overprotection (paternal: *r* = 0.397; maternal: *r* = 0.506), and generalized anxiety levels (*r* = 0.291), and significantly and negatively correlated with parental emotional warmth (paternal: *r* = −0.212; maternal: *r* = −0.239). All correlation coefficients were significant at *p* < 0.001, indicating satisfactory criterion-related validity of the scale.

#### 3.1.6. Reliability.

The Cronbach’s α coefficient was 0.922 for the full scale, ranging from 0.741 to 0.912 for the three sub-dimensions. The 6-week test-retest ICC was 0.825 for the total scale, with sub-dimension values ranging from 0.604 to 0.849.

#### 3.1.7. Measurement invariance across gender.

Multi-group CFA was conducted to examine measurement invariance across gender. The results showed that changes in fit indices across successive models met the recommended criteria (|ΔCFI | < 0.010, |ΔRMSEA| < 0.015) [58], supporting configural, metric, and scalar invariance (Table 4). These findings indicated that the PPEAS measures the same construct for male and female participants.

**Table 4:** Testing for measurement invariance across gender(n = 287).

Model	χ²/df	CFI	TLI	RMSEA	ΔCFI	ΔRMSEA
Configural invariance	2.061	0.914	0.897	0.061	–	–
Metric invariance	2.025	0.911	0.900	0.060	−0.003	−0.001
Scalar invariance	2.024	0.905	0.900	0.060	−0.006	0.000

### 3.2. Influencing factors of PPEA

#### 3.2.1. Level and demographic characteristics of PPEA among Chinese college freshmen.

Among the 646 college freshmen, the median total score of PPEA was 40.00 (IQR: 32.00, 49.00). Higher PPEA levels were associated with male gender, being an only-child, having divorced parents, having authoritarian parents, and having at least one parent supervising academic (Table 5).

**Table 5:** Level of PPEA and its demographic characteristics in college freshmen (n = 646).

Variables	Frequency	Scores of PPEA *M(P25, P75)*	*Z/H*	*P*
Total score	646	40.00(32.00, 49.00)		
Gender				
Male	415(64.2%)	42.00(32.00, 50.00)	−2.414	**0.016**
Female	231(35.8%)	37.00(30.00, 48.00)		
Age			−0.528	0.560
≤19 years	493(76.3%)	40.00(32.00, 50.00)		
>19 years	153(23.7%)	40.00(31.00, 48.00)		
Parental Marital Status			−2.273	**0.023**
Not divorced	571(88.4%)	38.00(31.00, 48.00)		
Divorced	75(11.6%)	44.00(34.00, 52.00)		
Only child			−2.257	**0.024**
Yes	215(33.3%)	42.00(33.00, 50.00)		
No	431(66.7%)	38.00(30.00, 48.00)		
Paternal education level			0.005	0.998
University	269(41.6%)	39.00(31.00, 50.00)		
Senior high school	159(24.6%)	41.00(32.00, 49.00)		
Junior high school or below	218(33.7%)	40.00(32.00, 48.00)		
Maternal education level			0.121	0.941
University	184(28.5%)	38.00(30.25, 50.00)		
Senior high school	188(29.1%)	40.50(32.00, 50.00)		
Junior high school or below	274(42.4%)	40.00(32.00, 48.00)		
Family economic status			1.612	0.447
Poor	55(8.5%)	40.00(32.00, 49.00)		
Average	564(87.3%)	40.00(32.00, 49.00)		
Well-off	27(4.2%)	36.00(28.00, 48.00)		
Parenting Style			81.665	**<0.001**
Authoritarian	100(15.5%)	52.50(46.00, 65.00)		
Permissive	81(12.5%)	33.00(26.50, 46.00)		
Indulgent	21(3.3%)	42.00(24.50, 49.50)		
Democratic	444(68.7%)	37.50(31.25, 47.00)		
Primary parents managing study				
Neither parent	170(26.3%)	34.00(26.00, 44.00)	28.799	**<0.001**
Father	66(10.2%)	40.00(29.00, 49.25)		
Mother	223(34.5%)	42.00(33.00, 52.00)		
Both parents	187(28.9%)	42.00(33.00, 51.00)		

Notes:*p < 0.05. **p < 0.01. ***p < 0.001.

#### 3.2.2. Correlations between family conflict, cohesion, children’s resilience and PPEA.

Spearman correlation analyses showed that PPEA was negatively correlated with family cohesion (*r* = −0.242, *p* < 0.001) and resilience (*r* = −0.194, *p* < 0.001), and positively correlated with family conflict (*r* = 0.262, *p* < 0.001).

#### 3.2.3. Predictors of PPEA.

Hierarchical linear regression was performed with PPEA as the dependent variable. Model 1 included demographic variables; Model 2 further added family variables; children’s resilience was entered into Model 3 (Table 6). Prior to hierarchical regression, we examined all key assumptions of linear regression. As indicated by the Shapiro-Wilk test, the residuals were normally distributed (*p* = 0.200). Moreover, the residual scatter plot showed that the assumptions of linearity and homoscedasticity were satisfied. All VIFs were below 5, indicating no obvious multicollinearity. The Durbin-Watson statistic was 1.938, falling within the acceptable range of 1.5 to 2.5, thereby supporting independent error terms. The maximum Cook’s distance was 0.01, far below the critical cutoff of 1.0, indicating no influential outliers. These results verified that the assumptions for linear regression were sufficiently satisfied.

**Table 6:** Hierarchical linear regression for PPEA (n = 646).

Variables	Model 1		Model2		Model3	
	β	t	β	t	β	t
**Socio-demographic variables**						
Gender (Ref: female)						
Male	0.072	1.818	0.094	2.517*	0.115	3.073**
Age	−0.036	−0.892	−0.023	−0.638	−0.017	−0.471
Parental marital status (Ref: not divorced)						
Divorced	0.097	2.440*	0.053	1.461	0.051	1.418
Only child (Ref: no)						
Yes	0.084	2.059*	0.063	1.706	0.056	1.530
Paternal education level (Ref: university)						
Senior high school	−0.011	−0.226	0.002	0.046	0.004	0.089
Junior high school or below	−0.004	−0.070	−0.014	−0.255	−0.016	−0.292
Maternal education level (Ref: university)						
Senior high school	0.025	0.498	0.020	0.429	0.021	0.465
Junior high school or below	0.038	0.567	0.039	0.642	0.045	0.756
Family economic status (Ref: poor)						
Average	−0.027	−0.565	0.020	0.450	0.023	0.525
Well-off	−0.064	−1.323	−0.025	−0.553	−0.005	−0.111
**Family variables**						
Parental parenting styles (Ref: democratic)						
Authoritarian			0.282	7.350***	0.274	7.198***
Permissive			0.001	0.018	0.004	0.110
Indulgent			−0.011	−0.299	−0.006	−0.180
Cohesion			−0.109	−2.511*	−0.084	−1.910
Conflict			0.152	3.411***	0.136	3.053**
Primary academic supervisor (Ref: neither parent)						
Father			0.080	1.969*	0.080	1.989*
Mother			0.205	4.464***	0.216	4.739***
Both parents			0.162	3.550***	0.185	4.064***
**Resilience**					−0.138	−3.548***
**PPEA**						
**R** ^**2**^	0.026		0.227		0.242	
**Adjusted R** ^**2**^	0.011		0.205		0.219	
**F**	1.697		10.237***		10.540***	

Notes: *p < 0.05. **p < 0.01. ***p < 0.001; β in the table denotes standardized partial regression coefficients.

The overall model explained 24.2% of the total variance in PPEA. Demographic variables, family variables, and resilience accounted for 2.6%, 20.1%, and 1.5% of the variance, respectively. In the final model, six variables were significantly linked to higher PPEA: male gender, family conflict, authoritarian parenting style, and having at least one parent as the primary academic supervisor. By contrast, children’s resilience was associated with lower PPEA.

## 4. Discussion

Based on family system theory, this study revised the PEAS and formed PPEAS. The scale exhibited good reliability and validity and can serve as a scientific instrument to measure children’s retrospective PPEA. Furthermore, this study systematically observed the levels and influencing factors of PPEA among Chinese college freshmen.

### 4.1. The revised scale has good reliability and validity with a reasonable structure

The three retained factors of the revised PPEAS are perceived academic performance anxiety, perceived competitive environment anxiety, and perceived educational efficacy anxiety. This structure is consistent with the original scale, which was developed via systematic qualitative and quantitative research identifying these identical three core domains [20]. CFA supported the three-factor model, and the correlations among factors provide evidence for the scale’s construct validity, indicating that these three factors are not isolated but interrelated. An intense competitive environment raises parental attention toward children’s academic performance [4]. Parents with low educational efficacy are more vulnerable to pressures from the external competitive environment [59]. In addition, children’s unsatisfactory academic performance brings more negative feedback to parents, further undermining parents’ confidence in their educational capabilities [60]. Within a family system, members’ emotions mutually influence one another [10]; children actively perceive parental educational anxiety in specific scenarios such as receiving poor grades or discussing academic matters [20,61]. Accordingly, when children report their perceptions, their ratings across these three domains tend to correlate with one another, reflecting the interconnected anxiety cues they have perceived. Thus, the content of children’s perceptions matches the domains through which parents express educational anxiety, suggesting that the three-factor structure can adequately capture PPEA. Moreover, the scale showed satisfactory internal consistency reliability and test-retest reliability. In summary, the revised PPEAS has favorable structural validity and functions as a reliable and valid tool for measuring children’s PPEA.

### 4.2. Chinese college freshmen reported a relatively low level of PPEA, with higher score in male

The median PPEA score reported by freshmen in the current study was 40.00 (5-point scale; total score range: 16–80), which was numerically lower than the mean scores (46.27–50.77) reported by parents using the identical 5-point scale [20]. In addition, another study reported an average item score of 2.79 based on a 5-point scale [62], which exceeded the median item score of 2.50found in our sample. Although direct statistical comparisons cannot be conducted, these differences reveal a trend: children’s perceptions of parental anxiety may be lower than parents’ self-reporting. This discrepancy can be interpreted from the perspective of family system theory and construal-level theory of psychological distance (CLT). Within the family system, children have limited access to their parents’ internal thoughts and affective states, resulting in divergent perceptions of parental anxiety [63]. Furthermore, the retrospective design of the present study introduces substantial temporal distance. According to CLT, such distance renders memories more abstract [64]. Specifically, vivid and concrete details related to parental anxiety (e.g., specific interactions, urgent tones) may fade in memory and be reconstructed into abstract concerns (e.g., “*my parents cared about my future*”) [64], which may cause children to perceive parental anxiety as less intense than what parents actually experienced. This highlights a mismatch between PPEA and parental self-reported educational anxiety and underscores the necessity of measuring children’s perception to understand its unique impact on children’s mental health.

Moreover, males reported higher PPEA levels. This phenomenon can be explained within the Chinese socio-cultural context. Chinese parents often hold higher educational expectations for boys than for girls [65]. Accordingly, baseline expectations are set higher for boys. When children’s academic performance fails to meet parental expectations, the resulting “expectation gap” may amplify parental educational anxiety, which can be transmitted to children [7]. These results suggest that parents of boys should avoid excessive expectations, as such expectations not only elevate their own educational anxiety but also place pressure on their children. This insight highlights the value of establishing diverse and reasonable criteria for success to curb the excessive transmission of educational anxiety.

### 4.3. Influences of family conflict, family cohesion, and children’s resilience on PPEA

Although the PPEA scores were non-normally distributed, the hierarchical regression results were robust, as all diagnostic assumptions (normality of residuals, linearity, homoscedasticity, no multicollinearity, and no influential outliers) were met. Hierarchical regression revealed that higher PPEA was associated with greater family conflict and lower levels of children’s resilience, whereas family cohesion was not a significant predictor. These findings partially support our hypotheses. The positive association between family conflict and PPEA can be interpreted from two perspectives. On the one hand, family conflict may elevate parents’ own anxiety. Existing studies have confirmed that family conflict correlates with elevated anxiety and depression [66], and the family system perspective indicates that such anxiety can spill over into educational interactions [10], thereby increasing children’s PPEA. On the other hand, family conflict undermines children’s emotional security and heightens their vigilance toward parental negative emotional cues, rendering them more sensitive to parental educational anxiety [24,67]. Nevertheless, contrary to our hypothesis, family cohesion did not independently predict PPEA in the final model. A possible explanation is that the effect size of cohesion was relatively small, and its independent contribution was no longer significant when stronger predictors such as family conflict and authoritarian parenting style were simultaneously entered into the model. At the individual level, children’s resilience shows a negative association with their PPEA, which can be explained by the cognitive mechanism of selective perception within Kumpfer’s resilience framework [68]. Children with higher resilience may be better at rationally attributing their parents’ anxious behaviors and mobilizing more positive emotions and coping strategies to “filter” the perceived anxiety signals. Accordingly, the intensity of external stressors on their internal world can be significantly reduced [29,30]. As indicated by existing research, highly resilient individuals possess a great capacity for cognitive reappraisal [31], which may enable them to reinterpret their parents’ educational anxiety as a form of involved concern rather than a threatening signal. Thus, lower PPEA may be achieved. In addition, authoritarian parenting style also shows a significant correlation with higher PPEA. This may be because authoritarian parenting style serves as a primary behavioral conduit through which parents’ internal anxiety is expressed [69,70]. Children interpret these high-pressure, non-responsive interaction patterns as intense educational anxiety [69]. Together, the results suggest that both school and family education should focus on fostering harmonious family environment and enhancing children’s resilience to resist transmission of anxiety. Nevertheless, the final model explained only 24.2% of the variance in PPEA, indicating that substantial portion remains unexplained such as parent-child communication [28].

### 4.4. Limitations

Several limitations of this study need to be considered. First, samples exhibited substantial differences in gender distribution due to convenience sampling. In addition, as we excluded participants with current or history of mental disorders, substance abuse, or severe physical diseases, our findings are generalizable specifically for healthy college freshmen without these conditions, and the reliance on self-report may not capture undiagnosed cases. Future studies are recommended to adopt multicenter designs and gender-balanced samples to verify these findings. Second, the cross-sectional design precludes causal inferences. Longitudinal studies are needed to explore the causal relationships between these factors and PPEA. Third, as with retrospective self-report measures, participants’ current mood states may influence their responses [71]. Given the nature of the measure, current parent-child relationship quality may similarly shape their retrospective perceptions. Finally, we did not assess educational anxiety experienced by parents directly, and the criterion-related validity evidence for the PPEAS remains indirect. In future, parent-child dyad designs should be conducted to evaluate parent-child discrepancy and offer criterion validity of the PPEAS directly. Nevertheless, the current study offers a tool to access the perceived parental-educational-anxiety from children’s perspective and further observe the level and predictors of PPEA in Chinese college students.

## 5. Conclusion

This study revised and validated the PPEAS, providing a reliable instrument for assessing children’s PPEA. The findings indicated that family conflict was positively associated with PPEA, whereas children’s resilience was negatively associated with PPEA. Moreover, the level of PPEA reported by college freshmen appears lower than that previously reported by parents, suggesting a meaningful perception gap that warrants further parent-child pairing designs. These results highlight the importance of decreasing family conflict and enhancing children’s resilience to mitigate PPEA.

## Supporting information

S1 Table Perceived Parental Educational Anxiety Scale (PPEAS). (DOCX)

S1 File Dataset. (ZIP)
