# Supplementary material for: Revision of the perceived parental-educational-anxiety scale and its application among Chinese college freshmen
Source: PLoS One. 2026 Sep 16;21(9):e0358097. doi: 10.1371/journal.pone.0358097 (PMC13580987; doi:10.1371/journal.pone.0358097)
Supplement: Supplementary file 1 [file pone.0358097.s003.pdf]

Reviewer Comments

Recommendation: Major Revision

Overall Assessment

This manuscript addresses an important and timely topic by revising a measure of parental educational anxiety from the perspective of children and examining factors associated with perceived parental educational anxiety (PPEA) among Chinese college freshmen. The topic is relevant and potentially meaningful given increasing scholarly attention to educational anxiety, family processes, and student mental health. The use of separate samples for exploratory and confirmatory factor analyses is a strength, as is the attempt to establish multiple forms of validity and reliability.

However, the manuscript's primary contribution is the development and validation of a revised instrument. Therefore, the rigor of the scale adaptation process and the evidence supporting validity are critically important. Several methodological, psychometric, reporting, and interpretive concerns should be addressed before the manuscript can be considered for publication. Although the study demonstrates promise, substantial revisions are needed to strengthen the validity of the conclusions and improve alignment with PLOS ONE publication standards.

Major Comments

1. The scale revision process requires stronger justification and evidence of content validity.

The central contribution of the manuscript is the revision of the Parental Educational Anxiety Scale (PEAS) into the Perceived Parental Educational Anxiety Scale (PPEAS). However, the revision process appears to consist primarily of pronoun conversion (e.g., changing "I feel

anxious" to "My parents feel anxious"), review by one professor and nine graduate students, and interviews with five freshmen.

While this process represents an initial adaptation effort, it is unclear whether simple linguistic modification is sufficient to transform a parent self-report measure into a child-perception measure. Children may perceive parental educational anxiety differently than parents experience or report it. Consequently, stronger evidence of content validity is needed. The authors should provide a more detailed description of:

• The theoretical rationale for treating the revised measure as a distinct construct.

• The specific procedures used during expert review.

• Whether experts independently evaluated item relevance and clarity.

• Whether any formal content validity procedures (e.g., Content Validity Index) were conducted.

• The nature of the interviews conducted with the five freshmen and how feedback informed item revisions.

Because the instrument is the manuscript's primary contribution, the scale development process requires greater methodological transparency.

2. Criterion validity evidence is indirect and should be discussed more carefully.

Criterion-related validity was assessed using generalized anxiety and parenting style measures. While the reported correlations are theoretically reasonable, none of these instruments directly assess parental educational anxiety.

The manuscript repeatedly argues that children's perceptions differ from parental self-reports and that existing parental educational anxiety measures are inadequate for assessing PPEA. However, no parent-reported educational anxiety data were collected. As a result, the criterion validity evidence remains indirect.

The authors should acknowledge more explicitly that the study does not establish agreement or divergence between actual parental educational anxiety and children's perceptions. Future research should employ paired parent-child designs to evaluate convergent and discriminant validity more directly.

3. Potential common method bias should be addressed.

Because key study variables were collected through self-report questionnaires from the same respondents, including PPEAS, family cohesion/conflict, resilience, and criterion-related measures such as generalized anxiety, the possibility of common method variance should be addressed.

4. Additional information regarding regression assumptions is needed.

Hierarchical multiple linear regression was used to identify factors associated with PPEA. However, the manuscript does not clearly report whether regression assumptions were evaluated. Please clarify whether the following assumptions were assessed:

- Linearity
- Independence of errors
- Multicollinearity
- Homoscedasticity
- Influential outliers
- Normality of residuals

This information is necessary to evaluate the technical rigor of the analyses.

5. Use of linear regression despite non-normal outcome distribution requires justification.

The authors report PPEA scores using medians and interquartile ranges because the data were non-normally distributed. However, the subsequent analyses rely on hierarchical linear regression.

The manuscript should explain why linear regression was considered appropriate despite the non-normal distribution of the outcome variable. If assumptions were met through examination of residuals, this should be reported explicitly. Alternatively, the authors should discuss the robustness of their findings.

6. Interpretation of findings occasionally exceeds what can be supported by a cross-sectional design.

Throughout the manuscript, terms such as "risk factor" and "protective factor" are used to describe family conflict and resilience.

Because the study is cross-sectional, causality cannot be established. Therefore, language implying causal influence should be avoided.

Terms such as:

• Associated with

• Related to

• Predictor of

would be more appropriate than:

• Risk factor

• Protective factor

• Increasing

• Reducing

unless supported by longitudinal evidence.

7. Retrospective recall bias represents an important limitation.

Participants were college freshmen reporting retrospectively on perceptions developed throughout their K-12 educational experiences.

Retrospective reporting introduces potential recall bias and reconstruction of memories. Current emotional functioning, current relationships with parents, and passage of time may influence responses.

Although the authors discuss retrospective design and temporal distance, the limitation section should more directly address recall bias and the possibility that current emotional functioning or current parent-child relationships shaped participants' responses.

8. Sampling procedures and sample characteristics require clarification.

The validation samples differ considerably in gender composition.

Sample 1 contained 31 females among 232 participants, whereas Sample 2 contained 177 females among 287 participants.

The authors should explain:

• Recruitment procedures for each sample.

• Reasons for the substantial differences in gender distribution.

• Whether gender imbalance may have influenced psychometric findings.

9. Data availability statements are inconsistent and may not satisfy PLOS ONE requirements.

The submission materials indicate that all data are available, while the manuscript states that data are available from the corresponding author upon request.

These statements are inconsistent.

PLOS ONE requires clear and transparent data availability statements. The authors should clarify:

• Whether the data are publicly available.

• Where the data can be accessed.

• Whether ethical or legal restrictions prevent public release.

The current wording does not appear sufficient.

Minor Comments

1. Abstract contains a likely typographical error.

The abstract indicates that Sample 2 was used for confirmatory factor analysis (EFA). This should likely be revised to CFA. The abstract should clearly distinguish EFA results from CFA model-fit results.

2. CFA model modification requires additional justification.

The manuscript reports adding a residual covariance between items 12 and 13 based on modification indices.

Although this improved model fit, the authors should provide a stronger theoretical explanation for allowing correlated errors and discuss any implications for construct validity.

3. Table 1 dummy coding should be clarified.

The coding scheme for the primary academic supervisor variable is difficult to interpret. Additional explanation would improve transparency and reproducibility.

4. Limited explained variance should be acknowledged.

The final regression model explains approximately 24.2% of the variance in PPEA.

While acceptable for psychosocial research, this indicates that a substantial proportion of variance remains unexplained. The authors should discuss additional factors that may influence PPEA.

5. Exclusion criteria require clarification.

Participants with current or past mental disorders, substance abuse, or severe physical illness were excluded.

The authors should clarify:

- How these conditions were assessed.
- Whether exclusions were based on self-report or clinical records.
- How these exclusions may affect generalizability.

6. English language editing is recommended.

Although the manuscript is understandable, there are numerous grammatical and stylistic issues throughout the text.

Examples include:

- "children's angle"
- "children's PPEA remains unknown, even lacks a standard tool"
- "all datas"
- "adequate requirement"

Professional English-language editing would improve readability and clarity.

Conclusion

This manuscript addresses an important topic and presents a potentially useful instrument for measuring perceived parental educational anxiety. The study has several strengths, including

adequate sample sizes, use of separate EFA and CFA samples, and multiple forms of psychometric evaluation.

However, because the manuscript's primary contribution is the development and validation of a revised scale, stronger evidence supporting the adaptation process, validity claims, statistical reporting, and interpretation of findings is needed. Clarification of data availability procedures and improved alignment between conclusions and study design are also necessary.

I therefore recommend Major Revision.
