# Supplementary material for: Revision of the perceived parental-educational-anxiety scale and its application among Chinese college freshmen
Source: PLoS One. 2026 Sep 16;21(9):e0358097. doi: 10.1371/journal.pone.0358097 (PMC13580987; doi:10.1371/journal.pone.0358097)
Supplement: Supplementary file 2 [file pone.0358097.s001.docx]

**S1. Perceived Parental Educational Anxiety Scale (PPEAS)**

**指导语**：下面是关于中小学阶段主要管你学习的父母在教育你时的焦虑感受的描述。请认真阅读以下每道题目，基于你的回忆和感知，从五个选项中勾选最符合你父母情况的一个。

**Instructions**: Please read each of the following statements which describe feelings of the parent who was primarily responsible for your learning in primary and secondary school stages. Based on your retrospective observation and perception, please indicate how frequently each situation occurred in your family by selecting the most appropriate option.

| **条目** | **Items** |
| --- | --- |
| **感知学业表现焦虑** | **Perceived academic performance anxiety** |
| 1. 如果发现我的学习方法比较低效，父母会很着急。 | 1. My parents would feel anxious if they found my learning methods were inefficient. |
| 2. 只要我的学习成绩有一点波动，父母就会感到惊慌。 | 2. My parents felt alarmed whenever there was any fluctuation in my grades. |
| 3. 父母会因为我的学习老是没有进步而烦躁。 | 3. My parents got agitated if I made no progress in my learning for a long time. |
| 4. 如果发现我学习不够主动，父母会很着急。 | 4. My parents would become anxious if they found I was not proactive in my studies. |
| 5.一想到我将来有可能上不了好学校或找不到好工作，父母就会感到担忧。 | 5. The thought that I might not get into a good school or find a good job in the future made my parents worried. |
| 6. 父母对我的学习状态不放心。 | 6. My parents felt uneasy about my learning status. |
| 7. 父母会因为我的学习表现达不到他们的期待而感到失落。 | 7. My parents felt disappointed when my academic performance failed to meet their expectations. |
| 8. 当父母发现我学习不够专注时，他们会很着急。 | 8. My parents would become anxious when they found I was not focused on my studies. |
| 9. 父母会担心我把太多时间浪费在学习以外的事情上，比如使用电子产品。 | 9. My parents worried that I spent too much time on non-academic activities, such as using electronic devices. |
| **感知竞争环境焦虑** | **Perceived competitive environment anxiety** |
| 10. 当身边的家长对孩子的教育很焦虑时，父母也会变得焦虑。 | 10. When other parents around them expressed anxiety about their children’s education, my parents also became anxious. |
| 11.看到教育方面的网络信息，例如升学政策解读、育儿建议，会让父母感到焦虑。 | 11. Information about education online (e.g., interpretations of enrollment policies, parenting advice) made my parents anxious. |
| 12. 当知道其他家长给孩子报辅导班，父母就会尽快给我也报上。 | 12. When they knew that other parents had signed their children up for tutoring, my parents quickly signed me up for similar classes. |
| 13. 父母会受媒体宣传的影响去给我争抢学习资源。 | 13. Influenced by media reports, my parents strived to secure educational resources for me. |
| **感知教育效能焦虑** | **Perceived educational efficacy anxiety** |
| 14. 父母觉得他们在教育我的时候还不够尽力。 | 14. My parents felt they were not trying hard enough in educating me. |
| 15. 父母不确定他们的教育方式对我来说是不是最好的。 | 15. My parents were uncertain whether their parenting approach was the best for me. |
| 16. 在教育我时，父母总会对自己的教育理念或教育方法产生怀疑。 | 16. My parents often doubted their educational philosophy or methods regarding my education. |

Notes: Response format: 1 = “*Never*”, 2 = “*Rarely*”, 3 = “*Sometimes*”, 4 = “*Often*”, 5 = “*Always*”.
