# Supplementary material for: Revision of the perceived parental-educational-anxiety scale and its application among Chinese college freshmen
Source: PLoS One. 2026 Sep 16;21(9):e0358097. doi: 10.1371/journal.pone.0358097 (PMC13580987; doi:10.1371/journal.pone.0358097)
Supplement: Supplementary file 4 [file pone.0358097.s005.docx]

**Dear Dr. Kumer Das,**

Thank you for giving us an opportunity to revise the manuscript. Please find the enclosed revised version of our manuscript entitled **“**Revision of the perceived parental-educational-anxiety scale and its application among Chinese college freshmen**”** for your consideration.

Many thanks for your constructive and detailed comments on the manuscript, which substantially improves the paper. In this revision version, we have addressed all of the comments made by the reviewers (see below, **line numbers are from the revised manuscript with revision marks**). With these changes, we hope that the manuscript will be of more relevance to the readership of your journal.

This manuscript is not in consideration elsewhere and the author reports no conflicts of interest. Moreover, the co-author has approved the revised version making significant contributions to the writing and conceptualization of the manuscript.

If I can be of any further assistance, please do not hesitate to contact me at my email address (daiqin101@hotmail.com). Thank you for your consideration.

Yours sincerely,

**Dai Qin**

**Response to Editor**

**Journal Requirements:**
When submitting your revision, we need you to address these additional requirements.
1. Please ensure that your manuscript meets PLOS ONE's style requirements, including those for file naming. The PLOS ONE style templates can be found at

[https://journals.plos.org/plosone/s/file?id=wjVg/PLOSOne_formatting_sample_main_body.pdf](https://track.editorialmanager.com/CL0/https:%2F%2Fjournals.plos.org%2Fplosone%2Fs%2Ffile%3Fid=wjVg%2FPLOSOne_formatting_sample_main_body.pdf/1/010f019f59489983-d117a1d2-5ff5-4623-b984-d06002653864-000000/tTvMfYkmtKo7-eJQJYkwjKl6jMR1Lca9_K4LS6Lyveo=258)

and[https://journals.plos.org/plosone/s/file?id=ba62/PLOSOne_formatting_sample_title_authors_affiliations.pdf](https://track.editorialmanager.com/CL0/https:%2F%2Fjournals.plos.org%2Fplosone%2Fs%2Ffile%3Fid=ba62%2FPLOSOne_formatting_sample_title_authors_affiliations.pdf/1/010f019f59489983-d117a1d2-5ff5-4623-b984-d06002653864-000000/8cEtrqmWMjOPIg4dVbd6u4L4Ec05tbgoQ8tGWlISdo8=258)

**Response:** Thank you for your reminder. We have carefully revised the manuscript formatting, file naming, and other stylistic details in accordance with PLOS ONE’s style requirements.

2. Your ethics statement should only appear in the Methods section of your manuscript. If your ethics statement is written in any section besides the Methods, please delete it from any other section.

**Response:** Thank you for this reminder. We have checked and confirmed that the ethics statement appears only in the Methods section.

3. Please provide a complete Data Availability Statement in the submission form, ensuring you include all necessary access information or a reason for why you are unable to make your data freely accessible. If your research concerns only data provided within your submission, please write "All data are in the manuscript and/or supporting information files" as your Data Availability Statement.

**Response:** Thank you for this suggestion. In accordance with PLOS ONE’s data policy, we have revised the Data Availability Statement in the manuscript to: “*Data analyzed in this study were available from the supplementary materials (Supplementary 2: dataset).*”

4. We note that the grant information you provided in the ‘Funding Information’ and ‘Financial Disclosure’ sections do not match.When you resubmit, please ensure that you provide the correct grant numbers for the awards you received for your study in the ‘Funding Information’ section.

**Response:** We apologize for this oversight. We have carefully checked the funding information and ensured that the grant numbers in both the ‘Funding Information’ section of the submission system and the ‘Funding’ section of the manuscript are now identical. The correct funding details are: Social Science Foundation of Chongqing (2023NDYB94), the Education Project of Army Medical University (2023yjsB02), and Natural Science Funding of Chongqing (CSTB2025NSCQ-GPX0065).

5. If the reviewer comments include a recommendation to cite specific previously published works, please review and evaluate these publications to determine whether they are relevant and should be cited. There is no requirement to cite these works unless the editor has indicated otherwise.

**Response:** We have reviewed the recommended publications and have cited those that are relevant to our study.

6. Please review your reference list to ensure that it is complete and correct. If you have cited papers that have been retracted, please include the rationale for doing so in the manuscript text, or remove these references and replace them with relevant current references. Any changes to the reference list should be mentioned in the rebuttal letter that accompanies your revised manuscript. If you need to cite a retracted article, indicate the article’s retracted status in the References list and also include a citation and full reference for the retraction notice.

**Response:**We have carefully reviewed the entire reference list and confirmed that all references are complete, correct, and none have been retracted.

**Additional Editor Comments :**

**Comment 1:** Even though the manuscript studies parental anxiety, a little more literature reviews would be helpful, where the following studies can be used: Khoshlessan and Das, 2017; Tang et al. (2022)

**Response:** We thank the editor for this suggestion. We have incorporated the two recommended references into the opening paragraph of the Introduction: “*In recent years, a growing body of research has examined education-related anxiety, including students’ academic anxiety and parental educational anxiety [1,2]. The latter has become a notable concern worldwide, particularly in East Asian contexts such as China [3].*”

(See Line 43– 45 in the first paragraph of Section “1. Introduction”)

**Comment 2:** The use of hierarchical regression is reasonable; however, regression analysis for data containing high leverage points cane be discussed in the literature review (See Dey, et al. (2015) for more information).

**Response:** We greatly appreciate this methodological suggestion. We have carefully read and cited the reference you recommended (See Line 203 – 205 in the first paragraph of Section “2.5. Statistical analysis”): “*Diagnostic statistics were examined in accordance with standard regression diagnostic procedures, with the aim of detecting potential influential observations (e.g., high leverage points) [55].*” Accordingly, we showed the results of diagnostic statistics in the Results section (See Line 280– 283 in the first paragraph of Section “3.2.3. Predictors of PPEA”): “*All VIFs were below 5, indicating no obvious multicollinearity. The Durbin-Watson statistic was 1.938, falling within the acceptable range of 1.5 to 2.5, thereby supporting independent error terms. The maximum Cook’s distance was 0.01, far below the critical cutoff of 1.0, indicating no influential outliers. These results verified that the assumptions for linear regression were sufficiently satisfied.*” We believe this addition strengthens the methodological transparency of our regression analyses.

**Response to Reviewer 1**

**Comment 1:** Assessment of Technical Soundness: Based on the information provided, the study appears technically sound overall. The authors used separate samples to develop and validate the revised scale and provided evidence that the instrument is both reliable and consistent. The statistical analyses appear appropriate for addressing the study objectives, and the conclusions are well supported by the data and findings presented.

**Comment 2:** The statistical methods appear appropriate and rigorous for addressing the study objectives, including the use of exploratory and confirmatory factor analyses (EFA/CFA) for scale validation, reliability analyses, non-parametric tests, and hierarchical regression. The authors used separate samples to develop and validate the revised scale, reported strong reliability statistics (Cronbach's α = 0.922; ICC = 0.825), and employed an adequate sample size to examine factors associated with PPEA.

The authors demonstrate a thoughtful and appropriate interpretation of their findings throughout the manuscript. The discussion and conclusions are well aligned with the study design, statistical analyses, and the cross-sectional nature of the data. The discussion remains focused on associations supported by the findings, and the authors avoid overstating causal relationships. In particular, the use of the terms "risk factor" and "protective factor" is appropriate and reflects observed associations rather than causal claims, resulting in a balanced and scientifically sound interpretation of the results. The conclusions are generally consistent with the findings, including the associations between family conflict and higher PPEA and between resilience and lower PPEA.

**Response:** We sincerely thank you for your thorough and positive evaluation of our statistical approach. We are encouraged by your recognition that our interpretation remains well aligned with the cross-sectional design.

**Comment 3:** Availability of Data: The manuscript includes the revised PPEAS instrument, coding information, and detailed summary tables describing the statistical analyses and findings. However, the Data Availability Statement indicates that the data are available from the corresponding author upon request. To align with the journal's data availability requirements, the authors may consider providing access to the underlying de-identified dataset by depositing the data in a public repository or providing them as supporting information. Making the data publicly accessible would strengthen the transparency of the study.

**Response:** Thank you for your detailed suggestion regarding data transparency. In response to this comment, we have prepared the de-identified dataset and uploaded it as supplementary materials (**S2_Dataset**). Accordingly, we have revised our data availability statement in the manuscript to “*Data analyzed in this study were available from the supplementary materials (Supplementary 2: dataset).*”

(See Line 406)

**Comment 4:** Manuscript Presentation, Organization, Language, and Writing Quality: The manuscript is generally understandable and well organized. The clear structure of the manuscript, consisting of the Introduction, Materials and Methods, Results, Discussion, and Conclusion sections, makes it easy to follow and enhances readability.

However, the manuscript contains numerous grammatical errors and non-standard English expressions. Careful language editing is recommended to improve clarity, readability, and adherence to standard academic English. Examples include:

A) Line 28: "for confirmatory factor analysis (EFA) and reliability testing" appears to contain a typographical error. "EFA" should be revised to "CFA" (Confirmatory Factor Analysis).

B) Line 23, 24: "This study aimed to revise the Parental Educational Anxiety Scale from parents' perspective to children's angle." Consider revising to: "This study aimed to adapt the Parental Educational Anxiety Scale from the parents' perspective to the children's perspective."

C) Line 29: "646 participants completed revised PPEAS..." Consider revising to: "646 participants completed the revised PPEAS..."

D) Line 39: "providing a reliable tool for measuring perceived educational anxiety from children's perspective." Consider revising to: "providing a reliable tool for measuring perceived parental educational anxiety from the children's perspective."

E) Line 81: "Moreover, PPEA may be influenced by some factors." Consider revising to: "Moreover, PPEA may be influenced by several factors."

F) Line 211: "The result showed suitable for factor analysis" Consider revising to: "The results showed that the data were suitable for factor analysis."

G) Line 232: Please revise the phrase "less paternal emotional warmth" to "lower parental emotional warmth" for clarity, as both paternal and maternal emotional warmth are reported in this section.

H) Line 277: Please revise "indicating these three factors are not isolate but interrelated." to "indicating these three factors are not isolated but interrelated."

I) Line 315, 316: The sentence contains a grammatical error. Please revise "which not only increase their own educational anxiety but also pressure on their children." to "which not only increase their own educational anxiety but also place pressure on their children."

J) Line 340 - 342: Please provide an appropriate citation to support the statement: "Moreover, prior research indicates that highly resilient individuals possess a great capacity for cognitive reappraisal, which may enable them to reinterpret their parents' educational anxiety as a form of involved concern rather than a threatening signal, thereby contributing to lower PPEA."

Overall, this manuscript addresses an important topic and has the potential to make a meaningful contribution to the literature. Addressing the concerns above would improve the clarity, transparency, and overall quality of the manuscript.

**Response:** We sincerely thank you for carefully identifying these specific language issues, and we have corrected all the highlighted errors point-by-point as follows:

A) The original phrase “for confirmatory factor analysis (EFA) and reliability testing” has been revised to “*for confirmatory factor analysis (CFA) and reliability testing*.”(See Line 28 in the Abstract

B) The original sentence “This study aimed to revise the Parental Educational Anxiety Scale from parents’ perspective to children’s angle.” has been revised to “*This study aimed to revise the Parental Educational Anxiety Scale from a parental perspective to a child-perceived perspective.*”(See Line 22 – 23 in the Abstract)

C) The original phrase “646 participants completed revised PPEAS...” has been revised to “*646 participants completed the revised PPEAS.*” (See Line 28 in the Abstract)

D) The original phrase “providing a reliable tool for measuring perceived educational anxiety from children's perspective” has been revised to “*providing a reliable instrument for measuring perceived educational anxiety from the children’s perspective*”.(See Line 37 – 38 in the Abstract)

E) The original sentence “Moreover, PPEA may be influenced by some factors.” has been revised to “*Moreover, PPEA may be influenced by several factors.*” (See Line 80 in the Introduction)

F) The original phrase “The result showed suitable for factor analysis” has been revised to “*The results confirmed the suitability of the data for factor analysis.*” (See Line 223 in the Results)

G) The original phrase “less paternal emotional warmth” has been revised to “*significantly and negatively correlated with parental emotional warmth*”.(See Line 248-249 in Section “3.1.5. Criterion-related validity”)

H) The original phrase “indicating these three factors are not isolate but interrelated” has been revised to “*indicating that these three factors are not isolated but interrelated*”.(See Line 303 – 304 in the first paragraph of Section “4.1. The revised scale has good reliability and validity with a reasonable structure”)

I) The original phrase “which not only increase their own educational anxiety but also pressure on their children.” has been revised to “*as such expectations not only elevate their own educational anxiety but also place pressure on their children.*” (See Line 340-341 in the second paragraph of Section “4.2. Chinese college freshmen reported a relatively low PPEA, with higher scores in males”)

J) Thank you for pointing out this missing reference. We have added a citation to support the statement regarding cognitive reappraisal in resilient individuals: “*As indicated by existing research, highly resilient individuals possess a great capacity for cognitive reappraisal [31], which may enable them to reinterpret their parents’ educational anxiety as a form of involved concern rather than a threatening signal. Thus,lower PPEA may be achieved.*”(See Line 364-367 in the first paragraph of Section “4.3. Influences of family conflict, family cohesion and children’s resilience on PPEA”)

Beyond addressing each specific issue you raised above, we have had the entire manuscript professionally copyedited to correct the grammatical errors and ensure language clarity and readability. The editing certificate has been listed here.

**Response to Reviewer 2**

**Major Comments**

**Comment 1:** The scale revision process requires stronger justification and evidence of content validity.

The central contribution of the manuscript is the revision of the Parental Educational Anxiety Scale (PEAS) into the Perceived Parental Educational Anxiety Scale (PPEAS). However, the revision process appears to consist primarily of pronoun conversion (e.g., changing "I feel

anxious" to "My parents feel anxious"), review by one professor and nine graduate students, and interviews with five freshmen.

While this process represents an initial adaptation effort, it is unclear whether simple linguistic modification is sufficient to transform a parent self-report measure into a child-perception measure. Children may perceive parental educational anxiety differently than parents experience or report it. Consequently, stronger evidence of content validity is needed. The authors should provide a more detailed description of:

• The theoretical rationale for treating the revised measure as a distinct construct.

• The specific procedures used during expert review.

• Whether experts independently evaluated item relevance and clarity.

• Whether any formal content validity procedures (e.g., Content Validity Index) were conducted. • The nature of the interviews conducted with the five freshmen and how feedback informed item revisions.

Because the instrument is the manuscript's primary contribution, the scale development process requires greater methodological transparency.

**Response:** We sincerely thank you for this valuable comment. We have added details as follows:

**(1) Sub-question 1:** “*First, items were converted from self-reporting style to children’s perspective (e.g., “I feel anxious” was converted to “My parents feel anxious”). To be specific, the assessment of parents’ internal experiences shifts to the assessment of children’s perceptions of these experiences.*”

(See Line 120 – 122 in the second paragraph of Section “2.2. Scale revision”)

**(2) Sub-question 2-4:** “*Next, the content validity of the adapted items was evaluated by 10 experts (one professor and nine doctoral candidates with professional experience in educational or psychology). These experts rated the relevance of each item independently to the intended construct on a 4-point Likert scale (1 = not relevant, 4 = highly relevant). The item-level Content Validity Index (I-CVI) values for the 16 items ranged from 0.90 to 1.00 (> 0.78). Moreover, the scale-level CVI (S-CVI) based on the universal agreement method reached 0.88 (>0.80). The above-mentioned results indicated good content validity of the draft scale.*”

(See Line 122 – 128 in the second paragraph of Section “2.2. Scale revision”)

**(3) Sub-question 5:** “*Finally, given that the revised scale was designed for college freshmen, a population in emerging adulthood, 5 college freshmen were recruited for semi-structured interviews to examine face validity and comprehensibility. The interview guide focused on participants’ comprehension of each item of PPEAS, as well as any supplement for perceived parental-educational-anxiety, identification of any ambiguous wording or new suggestion, and confirmation that the scale and items clearly measured perceived parental educational anxiety from a child-perceived perspective. Based on the feedback, item 15 was revised from “My parents were uncertain whether their current parenting approach was the best for me” to “My parents were uncertain whether their parenting approach was the best for me” to match the retrospective design. No other major modifications were conducted. The final 16-item perceived parental educational anxiety scale (PPEAS) was in* ***Table S1****.*”

(See Line 128 – 137 in the second paragraph of Section “2.2. Scale revision”)

**Comment 2:** Criterion validity evidence is indirect and should be discussed more carefully. Criterion-related validity was assessed using generalized anxiety and parenting style measures. While the reported correlations are theoretically reasonable, none of these instruments directly assess parental educational anxiety. The manuscript repeatedly argues that children's perceptions differ from parental self-reports and that existing parental educational anxiety measures are inadequate for assessing PPEA. However, no parent-reported educational anxiety data were collected. As a result, the criterion validity evidence remains indirect.

The authors should acknowledge more explicitly that the study does not establish agreement or divergence between actual parental educational anxiety and children's perceptions. Future research should employ paired parent-child designs to evaluate convergent and discriminant validity more directly.

**Response:** Thank you for the inspiring suggestion. We added this in the limitations: “*Finally, we did not assess educational anxiety experienced by parents directly, and the criterion-related validity evidence for the PPEAS remains indirect. In future, parent-child dyad designs should be conducted to evaluate parent-child discrepancy and offer criterion validity of the PPEAS directly.*”

(See Line 385-388 in the last paragraph of Section “4.4 limitations”)

**Comment 3:** Potential common method bias should be addressed.

Because key study variables were collected through self-report questionnaires from the same respondents, including PPEAS, family cohesion/conflict, resilience, and criterion-related measures such as generalized anxiety, the possibility of common method variance should be addressed.

**Response:** Thank you for the important suggestion and revised accordingly. We have added this information to the Statistical analysis section: “*Harman’s single-factor test was performed to evaluate potential common method bias. The first unrotated factor explained 27.709% of the total variance, which was lower than50% [56], indicating no obvious common method bias in this study.*”

(See Line 208-210 in the second paragraph of Section “2.5. Statistical analysis”)

**Comment 4:** Additional information regarding regression assumptions is needed.

Hierarchical multiple linear regression was used to identify factors associated with PPEA. However, the manuscript does not clearly report whether regression assumptions were evaluated. Please clarify whether the following assumptions were assessed:

• Linearity

• Independence of errors

• Multicollinearity

• Homoscedasticity

• Influential outliers

• Normality of residuals

This information is necessary to evaluate the technical rigor of the analyses.

**Comment 5:** Use of linear regression despite non-normal outcome distribution requires justification.

The authors report PPEA scores using medians and interquartile ranges because the data were non-normally distributed. However, the subsequent analyses rely on hierarchical linear regression. The manuscript should explain why linear regression was considered appropriate despite the non-normal distribution of the outcome variable. If assumptions were met through examination of residuals, this should be reported explicitly. Alternatively, the authors should discuss the robustness of their findings.

**Response to Comment 4 and 5:** Thank you for this important methodological advice. As comment 4 and 5 are related, we address them together. We have systematically evaluated all key assumptions of linear regression and added a consolidated statement: “*Prior to hierarchical regression, we examined all key assumptions of linear regression. As indicated by the Shapiro-Wilk test, the residuals were normally distributed (p = 0.200). Moreover, the residual scatter plot showed that the assumptions of linearity and homoscedasticity were satisfied. All VIFs were below 5, indicating no obvious multicollinearity. The Durbin-Watson statistic was 1.938, falling within the acceptable range of 1.5 to 2.5, thereby supporting independent error terms. The maximum Cook’s distance was 0.01, far below the critical cutoff of 1.0, indicating no influential outliers. These results verified that the assumptions for linear regression were sufficiently satisfied.*”

(See Line 277-283 in the first paragraph of Section “3.2.3.Predictors of PPEA”)

In addition, we have added a brief statement in the Discussion section to explicitly address the robustness of the findings: “*Although the PPEA scores were non-normally distributed, the hierarchical regression results were robust, as all diagnostic assumptions (normality of residuals, linearity, homoscedasticity, no multicollinearity, and no influential outliers) were met.*”

(See Line 345-347 in the first paragraph of Section “4.3. Influences of family conflict, family cohesion and children’s resilience on PPEA”)

**Comment 6:** Interpretation of findings occasionally exceeds what can be supported by a cross-sectional design.

Throughout the manuscript, terms such as "risk factor" and "protective factor" are used to describe family conflict and resilience. Because the study is cross-sectional, causality cannot be established. Therefore, language implying causal influence should be avoided.

Terms such as:

• Associated with

• Related to

• Predictor of

would be more appropriate than:

• Risk factor

• Protective factor

• Increasing

• Reducing

unless supported by longitudinal evidence.

**Response:** Thank you for this important methodological suggestion. We have systematically revised the manuscript to replace all causal terms (e.g., “risk factor”, “protective factor”, “increasing”, “reducing”) with descriptive, non-causal language (e.g., “positively associated with”, “negatively associated with”, “related to”). We have also revised the Conclusion to frame our recommendations more cautiously. All the changes are as follows:

Line 34 – 35: “*Hierarchical regression showed that family conflict was positively associated with PPEA, while children’s resilience was negatively associated with PPEA.*”

Line 289-290: “*By contrast, children’s resilience was associated with lower PPEA.*”

Line 367-368: “*In addition, authoritarian parenting style also shows a significant correlation with higher PPEA.*”

Line 393– 394: “*The findings indicated that family conflict was positively associated with PPEA, whereas children’s resilience was negatively associated with PPEA.*”

**Comment 7:** Retrospective recall bias represents an important limitation.

Participants were college freshmen reporting retrospectively on perceptions developed throughout their K-12 educational experiences. Retrospective reporting introduces potential recall bias and reconstruction of memories. Current emotional functioning, current relationships with parents, and passage of time may influence responses. Although the authors discuss retrospective design and temporal distance, the limitation section should more directly address recall bias and the possibility that current emotional functioning or current parent-child relationships shaped participants’ responses.

**Response:** We sincerely thank you for this thoughtful suggestion and revised accordingly in the Limitation section: “*Third, as with retrospective self-report measures, participants’ current mood states may influence their responses [71]. Given the nature of the measure, current parent-child relationship quality may similarly shape their retrospective perceptions.*” We hope this revision can address your concern.

(See Line 383-385 in the last paragraph of Section “4.4. Limitations”)

**Comment 8:** Sampling procedures and sample characteristics require clarification.

The validation samples differ considerably in gender composition. Sample 1 contained 31 females among 232 participants, whereas Sample 2 contained 177 females among 287 participants.

The authors should explain:

• Recruitment procedures for each sample.

• Reasons for the substantial differences in gender distribution.

• Whether gender imbalance may have influenced psychometric findings.

**Response:** Thank you for the important suggestion and revised accordingly.

(1) The recruitment procedures were described in detail in the manuscript: “*College freshmen from two universities in Chongqing, China, were recruited for this survey from April to July 2025. Convenience sampling was adopted to enroll participants through advertising from different departments.*”(See Line 101-103 in Section “2.1. Participants”)

“*Participants were recruited from two universities in Chongqing. Prior to the survey, the purpose, voluntary nature and confidentiality were clearly explained to potential participants, and written informed consent was obtained.*” (See Line 192-194 in Section “2.4. Procedure”)

(2) Reasons for the substantial differences in gender distribution: The substantial gender imbalance across samples was primarily an incidental outcome of class-based convenience sampling, reflecting the underlying gender composition of the respective academic disciplines. We have acknowledged this in limitation: “*First, samples exhibited substantial differences in gender distribution due to convenience sampling. In addition, as we excluded participants with current or history of mental disorders, substance abuse, or severe physical diseases, our findings are generalizable specifically for healthy college freshmen without these conditions, and the reliance on self-report may not capture undiagnosed cases. Future studies are recommended to adopt multicenter designs and gender-balanced samples to verify these findings.*”

(See Line 376-381 in Section “4.4. Limitations”)

(3) Whether gender imbalance may have influenced psychometric findings: To solve this problem, we conducted a multi-group confirmatory factor analysis to formally test measurement invariance across male and female participants using Sample 2: “*Multi-group CFA was conducted to examine measurement invariance across gender. The results showed that changes in fit indices across successive models met the recommended criteria (|ΔCFI | < 0.010, |ΔRMSEA|< 0.015) [58], supporting configural, metric, and scalar invariance (****Table 4****). These findings indicated that the PPEAS measures the same construct for male and female participants.*”

***Table 4 Testing for measurement invariance across gender (n = 287)***

| Model | χ²/df | CFI | TLI | RMSEA | ΔCFI | ΔRMSEA |
| --- | --- | --- | --- | --- | --- | --- |
| Configural invariance | 2.061 | 0.914 | 0.897 | 0.061 | – | – |
| Metric invariance | 2.025 | 0.911 | 0.900 | 0.060 | -0.003 | -0.001 |
| Scalar invariance | 2.024 | 0.905 | 0.900 | 0.060 | -0.006 | 0.000 |

(See Line 256-260 in Section “3.1.7. Measurement invariance across gender”)

**Comment 9:** Data availability statements are inconsistent and may not satisfy PLOS ONE requirements.

The submission materials indicate that all data are available, while the manuscript states that data are available from the corresponding author upon request. These statements are inconsistent.

PLOS ONE requires clear and transparent data availability statements. The authors should clarify: • Whether the data are publicly available.

• Where the data can be accessed.

• Whether ethical or legal restrictions prevent public release.

The current wording does not appear sufficient.

**Response:** Thank you for the suggestion and revised accordingly. We have updated the data availability statements as “*Data analyzed in this study were available from the supplementary materials (Supplementary 2: dataset).*”

**Minor Comments**

**Comment 1:** Abstract contains a likely typographical error. The abstract indicates that Sample 2 was used for confirmatory factor analysis (EFA). This should likely be revised to CFA. The abstract should clearly distinguish EFA results from CFA model-fit results.

**Response:** Thank you for the suggestion and revised accordingly. We have corrected “EFA” to “CFA”.

(See Line 28 in the Abstract)

**Comment 2:** CFA model modification requires additional justification. The manuscript reports adding a residual covariance between items 12 and 13 based on modification indices. Although this improved model fit, the authors should provide a stronger theoretical explanation for allowing correlated errors and discuss any implications for construct validity.

**Response:** Thank you for the important suggestion and revised accordingly. In the revised manuscript, we added the following justification: “*When the modification indices (MI) exceed 10 and the items share similar content and wording, residual correlation can be added between them [57]. Items 12 and 13 belong to same dimension and possess similar wording, with MI exceeding the recommended threshold, providing a reasonable theoretical basis for correlated measurement errors. Therefore, error covariance was conducted between Items 12 and 13.As this modification was limited to a single pair of the items and was theoretically grounded, the construct validity of the scale is unlikely to be compromised.*”

(See Line 230 – 235 in Section “3.1.3. Confirmatory Factor Analysis”)

**Comment** 3: Table 1 dummy coding should be clarified. The coding scheme for the primary academic supervisor variable is difficult to interpret. Additional explanation would improve transparency and reproducibility.

**Response:** We are sorry for our careless mistakes in Table 1. The coding for “Both parents” was incorrectly listed as (Z1 = 0, Z2 = 1, Z3 = 1); it should have been (Z1 = 0, Z2 = 0, Z3 = 1), with Z1 = father, Z2 = mother, and Z3 = both parents. We have corrected this in the revised Table 1(See Line 211).

**Comment** 4: Limited explained variance should be acknowledged.

The final regression model explains approximately 24.2% of the variance in PPEA. While acceptable for psychosocial research, this indicates that a substantial proportion of variance remains unexplained. The authors should discuss additional factors that may influence PPEA.

**Response:** Thank you for the valuable suggestion and revised accordingly. We have added a sentence to acknowledge this: “*Nevertheless, the final model explained only 24.2% of the variance in PPEA, indicating that substantial portion remains unexplained such as parent-child communication [28].*”

(See Line 372 – 374 in the second paragraph of Section “4.3. Influences of family conflict, family cohesion and children’s resilience on PPEA”)

**Comment** 5: Exclusion criteria require clarification.

Participants with current or past mental disorders, substance abuse, or severe physical illness were excluded.

The authors should clarify:

• How these conditions were assessed.

• Whether exclusions were based on self-report or clinical records.

• How these exclusions may affect generalizability.

**Response:** Thank you for the valuable suggestion and revised accordingly. We have revised the exclusion criteria description: “*Exclusion criteria from self-reported questions included: current or history of mental disorders (major depression, bipolar disorder, anxiety disorder, et al), substance or drug abuse, or severe physical disease.*” (See Line 104-106 in the first paragraph of Section “2.1. Participants”)

We have also added a corresponding limitation in the Discussion section noting that: “*In addition, as we excluded participants with current or history of mental disorders, substance abuse, or severe physical diseases, our findings are generalizable specifically for healthy college freshmen without these conditions, and the reliance on self-report may not capture undiagnosed cases.*”(See Line 377-380 in the last paragraph of Section “4.4. Limitations”)

**Comment** 6: English language editing is recommended.

Although the manuscript is understandable, there are numerous grammatical and stylistic issues throughout the text.

Examples include:

• "children's angle"

• "children's PPEA remains unknown, even lacks a standard tool"

• "all datas"

• "adequate requirement"

Professional English-language editing would improve readability and clarity.

**Response:** Thank you for the important suggestion and revised accordingly. We have corrected all the errors mentioned above. Beyond addressing each specific issue raised above, we have had the entire manuscript professionally copyedited to correct grammatical errors and ensure language clarity and readability. The editing certificate has been listed here.

**Thank you for your valuable comments and giving us an opportunity to revise our manuscript. We hope that we have addressed the comments satisfactorily in this revision. If there are other errors or further requests, please contact us by e-mail (**[daiqin101@hotmail.com](mailto:daiqin101@hotmail.com)**).**

**Finally, I would like to thank you for your excellent suggestions which are so helpful to enhancing our study.**
