# Supplementary material for: Revision of the perceived parental-educational-anxiety scale and its application among Chinese college freshmen
Source: PLoS One. 2026 Sep 16;21(9):e0358097. doi: 10.1371/journal.pone.0358097 (PMC13580987; doi:10.1371/journal.pone.0358097)
Supplement: Peer review history

# Peer review: Decision Letter 0

**DOI:** 10.1371/journal.pone.0358097.r001

**Authors:** Kumer Das

12 Jul 2026

Dear Dr. Dai,

Thank you for submitting your manuscript to PLOS ONE. After careful consideration, we feel that it has merit but does not fully meet PLOS ONE’s publication criteria as it currently stands. Therefore, we invite you to submit a revised version of the manuscript that addresses the points raised during the review process.

Please submit your revised manuscript by Aug 11 2026 11:59PM. If you will need more time than this to complete your revisions, please reply to this message or contact the journal office at plosone@plos.org. When you're ready to submit your revision, log on to https://www.editorialmanager.com/pone/ and select the 'Submissions Needing Revision' folder to locate your manuscript file.

- A letter that responds to each point raised by the academic editor and reviewer(s). You should upload this letter as a separate file labeled 'Response to Reviewers'.
- A marked-up copy of your manuscript that highlights changes made to the original version. You should upload this as a separate file labeled 'Revised Manuscript with Track Changes'.
- An unmarked version of your revised paper without tracked changes. You should upload this as a separate file labeled 'Manuscript'.

If you would like to make changes to your financial disclosure, please include your updated statement in your cover letter. Guidelines for resubmitting your figure files are available below the reviewer comments at the end of this letter.

If applicable, we recommend that you deposit your laboratory protocols in protocols.io to enhance the reproducibility of your results. Protocols.io assigns your protocol its own identifier (DOI) so that it can be cited independently in the future. For instructions see: https://journals.plos.org/plosone/s/submission-guidelines#loc-laboratory-protocols. Additionally, PLOS ONE offers an option for publishing peer-reviewed Lab Protocol articles, which describe protocols hosted on protocols.io. Read more information on sharing protocols at https://plos.org/protocols?utm_medium=editorial-email&utm_source=authorletters&utm_campaign=protocols.

As the corresponding author, your ORCID iD is verified in the submission system and will appear in the published article. PLOS supports the use of ORCID, and we encourage all coauthors to register for an ORCID iD and use it as well. Please encourage your coauthors to verify their ORCID iD within the submission system before final acceptance, as unverified ORCID iDs will not appear in the published article. *Only* the individual author can complete the verification step; PLOS staff *cannot* verify ORCID iDs on behalf of authors.

We look forward to receiving your revised manuscript.

Kind regards,

Kumer Das

Academic Editor

PLOS One

Journal Requirements:

When submitting your revision, we need you to address these additional requirements.

1. Please ensure that your manuscript meets PLOS ONE's style requirements, including those for file naming. The PLOS ONE style templates can be found at

https://journals.plos.org/plosone/s/file?id=wjVg/PLOSOne_formatting_sample_main_body.pdfand https://journals.plos.org/plosone/s/file?id=ba62/PLOSOne_formatting_sample_title_authors_affiliations.pdf

2. Your ethics statement should only appear in the Methods section of your manuscript. If your ethics statement is written in any section besides the Methods, please delete it from any other section.

3. Please provide a complete Data Availability Statement in the submission form, ensuring you include all necessary access information or a reason for why you are unable to make your data freely accessible. If your research concerns only data provided within your submission, please write "All data are in the manuscript and/or supporting information files" as your Data Availability Statement.

4. We note that the grant information you provided in the ‘Funding Information’ and ‘Financial Disclosure’ sections do not match.

When you resubmit, please ensure that you provide the correct grant numbers for the awards you received for your study in the ‘Funding Information’ section.

5. If the reviewer comments include a recommendation to cite specific previously published works, please review and evaluate these publications to determine whether they are relevant and should be cited. There is no requirement to cite these works unless the editor has indicated otherwise.

6. Please review your reference list to ensure that it is complete and correct. If you have cited papers that have been retracted, please include the rationale for doing so in the manuscript text, or remove these references and replace them with relevant current references. Any changes to the reference list should be mentioned in the rebuttal letter that accompanies your revised manuscript. If you need to cite a retracted article, indicate the article’s retracted status in the References list and also include a citation and full reference for the retraction notice.

Additional Editor Comments :

The manuscript addresses parental educational anxiety, which has become a grave concern nowadays due to multiple factors. In the post-Covid-era, the phenomenon has grown significantly across the world. The study proposes an interesting angle of the parental educational anxiety. I agree with the assessment of the two reviewers. Moreover, I would like to offer a few suggestions to the authors.

1) Even though the manuscript studies parental anxiety, a little more literature reviews would be helpful, where the following studies can be used: Khoshlessan and Das, 2017; Tang et al. (2022)

2) The use of hierarchical regression is reasonable; however, regression analysis for data containing high leverage points cane be discussed in the literature review (See Dey, et al. (2015) for more information).

References:

R. Khoshlessan and Das, K. (2017) "Analyzing international students’ study anxiety in higher education", Journal of International Students, 2017, 7, pp 311-328

Tang, L, Matt, J, Khoshlessan, R., Das, K., and Allard, C. (2022) "A quantitative study of undergraduate students’ anxiety", Journal of education and Learning, 11(5), pp 15-30

Dey, A., Hossain, Md., Das, K. (2015), "Regression analysis for data containing outliers and high leverage points,", Alabama Journal of Mathematics, 39, p 1-6

[Note: HTML markup is below. Please do not edit.]

Reviewers' comments:

Reviewer's Responses to Questions

**Comments to the Author**

1. Is the manuscript technically sound, and do the data support the conclusions?

Reviewer #1: Yes

Reviewer #2: No

**********

2. Has the statistical analysis been performed appropriately and rigorously?

Reviewer #1: Yes

Reviewer #2: No

**********

3. Have the authors made all data underlying the findings in their manuscript fully available?

The PLOS Data policy

Reviewer #1: No

Reviewer #2: No

**********

4. Is the manuscript presented in an intelligible fashion and written in standard English?

Reviewer #1: Yes

Reviewer #2: No

**********

Reviewer #1: This manuscript addresses an important topic by revising the Perceived Parental Educational Anxiety Scale (PPEAS) and examining factors associated with perceived parental educational anxiety among Chinese college freshmen. The study contributes to the literature by providing a measure from the children's perspective and investigating the roles of family environment (family conflict and family cohesion) and children's resilience.

Strengths of the manuscript include the use of separate samples for exploratory and confirmatory factor analyses, the reporting of reliability indices, and the use of hierarchical regression analyses to examine potential predictors of PPEA. The findings are generally presented clearly, and the conclusions are largely supported by the results.

I have several suggestions that may strengthen the manuscript:

1. Assessment of Technical Soundness:

Based on the information provided, the study appears technically sound overall. The authors used separate samples to develop and validate the revised scale and provided evidence that the instrument is both reliable and consistent. The statistical analyses appear appropriate for addressing the study objectives, and the conclusions are well supported by the data and findings presented.

2. Assessment of Statistical Analyses:

The statistical methods appear appropriate and rigorous for addressing the study objectives, including the use of exploratory and confirmatory factor analyses (EFA/CFA) for scale validation, reliability analyses, non-parametric tests, and hierarchical regression. The authors used separate samples to develop and validate the revised scale, reported strong reliability statistics (Cronbach's α = 0.922; ICC = 0.825), and employed an adequate sample size to examine factors associated with PPEA.

The authors demonstrate a thoughtful and appropriate interpretation of their findings throughout the manuscript. The discussion and conclusions are well aligned with the study design, statistical analyses, and the cross-sectional nature of the data. The discussion remains focused on associations supported by the findings, and the authors avoid overstating causal relationships. In particular, the use of the terms "risk factor" and "protective factor" is appropriate and reflects observed associations rather than causal claims, resulting in a balanced and scientifically sound interpretation of the results. The conclusions are generally consistent with the findings, including the associations between family conflict and higher PPEA and between resilience and lower PPEA.

3. Availability of Data:

The manuscript includes the revised PPEAS instrument, coding information, and detailed summary tables describing the statistical analyses and findings. However, the Data Availability Statement indicates that the data are available from the corresponding author upon request. To align with the journal's data availability requirements, the authors may consider providing access to the underlying de-identified dataset by depositing the data in a public repository or providing them as supporting information. Making the data publicly accessible would strengthen the transparency of the study.

4. Manuscript Presentation, Organization, Language, and Writing Quality:

The manuscript is generally understandable and well organized. The clear structure of the manuscript, consisting of the Introduction, Materials and Methods, Results, Discussion, and Conclusion sections, makes it easy to follow and enhances readability.

However, the manuscript contains numerous grammatical errors and non-standard English expressions. Careful language editing is recommended to improve clarity, readability, and adherence to standard academic English. Examples include:

A) Line 28: "for confirmatory factor analysis (EFA) and reliability testing" appears to contain a typographical error. "EFA" should be revised to "CFA" (Confirmatory Factor Analysis).

B) Line 23, 24: "This study aimed to revise the Parental Educational Anxiety Scale from parents' perspective to children's angle." Consider revising to: "This study aimed to adapt the Parental Educational Anxiety Scale from the parents' perspective to the children's perspective."

C) Line 29: "646 participants completed revised PPEAS..." Consider revising to: "646 participants completed the revised PPEAS..."

D) Line 39: "providing a reliable tool for measuring perceived educational anxiety from children's perspective." Consider revising to: "providing a reliable tool for measuring perceived parental educational anxiety from the children's perspective."

E) Line 81: "Moreover, PPEA may be influenced by some factors." Consider revising to: "Moreover, PPEA may be influenced by several factors."

F) Line 211: "The result showed suitable for factor analysis" Consider revising to: "The results showed that the data were suitable for factor analysis."

G) Line 232: Please revise the phrase "less paternal emotional warmth" to "lower parental emotional warmth" for clarity, as both paternal and maternal emotional warmth are reported in this section.

H) Line 277: Please revise "indicating these three factors are not isolate but interrelated." to "indicating these three factors are not isolated but interrelated."

I) Line 315, 316: The sentence contains a grammatical error. Please revise "which not only increase their own educational anxiety but also pressure on their children." to "which not only increase their own educational anxiety but also place pressure on their children."

J) Line 340 - 342: Please provide an appropriate citation to support the statement: "Moreover, prior research indicates that highly resilient individuals possess a great capacity for cognitive reappraisal, which may enable them to reinterpret their parents' educational anxiety as a form of involved concern rather than a threatening signal, thereby contributing to lower PPEA."

Overall, this manuscript addresses an important topic and has the potential to make a meaningful contribution to the literature. Addressing the concerns above would improve the clarity, transparency, and overall quality of the manuscript.

Reviewer #2: The manuscript addresses an important and timely topic related to parental educational anxiety, student perception, family processes, and student mental health. The study has several strengths, including the use of separate samples for exploratory and confirmatory factor analyses, adequate sample sizes, and an effort to examine multiple forms of reliability and validity. The revised scale has the potential to make a meaningful contribution; however, several issues should be addressed before the manuscript is suitable for publication.

I selected “No” for the technical soundness/data support question because the manuscript has promise, but several concerns need to be resolved before the conclusions can be fully supported. The primary contribution of the manuscript is the revision and validation of the PPEAS; however, the scale adaptation process needs stronger justification, clearer evidence of content validity, and more detail about expert review and student feedback.

I selected “No” for the statistical analysis question because the analyses require additional reporting and justification. In particular, the manuscript should clarify whether regression assumptions were evaluated, justify the use of hierarchical linear regression given the non-normal distribution of PPEA scores, and provide more detail regarding the CFA model modification.

I selected “No” for the data availability question because the data availability statements appear inconsistent. The submission materials indicate that all data are available, while the manuscript states that data are available from the corresponding author upon request. The authors should clarify whether the data are publicly available, where they can be accessed, or whether ethical/legal restrictions prevent public release.

I selected “No” for the standard English/intelligibility question because the manuscript is understandable overall, but it contains numerous grammatical and stylistic issues that should be corrected before publication. Professional English-language editing is recommended.

I do not identify concerns regarding dual publication or research ethics beyond the need for clearer reporting and stronger alignment with PLOS ONE standards. Overall, I recommend Major Revision.

**********

what does this mean?). If published, this will include your full peer review and any attached files.

If you choose “no”, your identity will remain anonymous but your review may still be made public.

**Do you want your identity to be public for this peer review?** For information about this choice, including consent withdrawal, please see our Privacy Policy

Reviewer #1: No

Reviewer #2: No

**********

[NOTE: If reviewer comments were submitted as an attachment file, they will be attached to this email and accessible via the submission site. Please log into your account, locate the manuscript record, and check for the action link "View Attachments". If this link does not appear, there are no attachment files.]

To ensure your figures meet our technical requirements, please review our figure guidelines: https://journals.plos.org/plosone/s/figures

You may also use PLOS’s free figure tool, NAAS, to help you prepare publication quality figures: https://journals.plos.org/plosone/s/figures#loc-tools-for-figure-preparation.

NAAS will assess whether your figures meet our technical requirements by comparing each figure against our figure specifications.

Attachment Submitted filename: PLOS - Manuscript Review.pdf

# Author comment: Author response to Decision Letter 1

**DOI:** 10.1371/journal.pone.0358097.r002

10 Aug 2026

Dear Dr. Kumer Das,

Thank you for giving us an opportunity to revise the manuscript. Please find the enclosed revised version of our manuscript entitled “Revision of the perceived parental-educational-anxiety scale and its application among Chinese college freshmen” for your consideration.

Many thanks for your constructive and detailed comments on the manuscript, which substantially improves the paper. In this revision version, we have addressed all of the comments made by the reviewers (see below, line numbers are from the revised manuscript with revision marks). With these changes, we hope that the manuscript will be of more relevance to the readership of your journal.

This manuscript is not in consideration elsewhere and the author reports no conflicts of interest. Moreover, the co-author has approved the revised version making significant contributions to the writing and conceptualization of the manuscript.

If I can be of any further assistance, please do not hesitate to contact me at my email address (daiqin101@hotmail.com). Thank you for your consideration.

Yours sincerely,

Dai Qin

Response to Editor

Journal Requirements:

When submitting your revision, we need you to address these additional requirements.

1. Please ensure that your manuscript meets PLOS ONE's style requirements, including those for file naming. The PLOS ONE style templates can be found at

https://journals.plos.org/plosone/s/file?id=wjVg/PLOSOne_formatting_sample_main_body.pdf

andhttps://journals.plos.org/plosone/s/file?id=ba62/PLOSOne_formatting_sample_title_authors_affiliations.pdf

Response: Thank you for your reminder. We have carefully revised the manuscript formatting, file naming, and other stylistic details in accordance with PLOS ONE’s style requirements.

2. Your ethics statement should only appear in the Methods section of your manuscript. If your ethics statement is written in any section besides the Methods, please delete it from any other section.

Response: Thank you for this reminder. We have checked and confirmed that the ethics statement appears only in the Methods section.

3. Please provide a complete Data Availability Statement in the submission form, ensuring you include all necessary access information or a reason for why you are unable to make your data freely accessible. If your research concerns only data provided within your submission, please write "All data are in the manuscript and/or supporting information files" as your Data Availability Statement.

Response: Thank you for this suggestion. In accordance with PLOS ONE’s data policy, we have revised the Data Availability Statement in the manuscript to: “Data analyzed in this study were available from the supplementary materials (Supplementary 2: dataset).”

4. We note that the grant information you provided in the ‘Funding Information’ and ‘Financial Disclosure’ sections do not match.When you resubmit, please ensure that you provide the correct grant numbers for the awards you received for your study in the ‘Funding Information’ section.

Response: We apologize for this oversight. We have carefully checked the funding information and ensured that the grant numbers in both the ‘Funding Information’ section of the submission system and the ‘Funding’ section of the manuscript are now identical. The correct funding details are: Social Science Foundation of Chongqing (2023NDYB94), the Education Project of Army Medical University (2023yjsB02), and Natural Science Funding of Chongqing (CSTB2025NSCQ-GPX0065).

5. If the reviewer comments include a recommendation to cite specific previously published works, please review and evaluate these publications to determine whether they are relevant and should be cited. There is no requirement to cite these works unless the editor has indicated otherwise.

Response: We have reviewed the recommended publications and have cited those that are relevant to our study.

6. Please review your reference list to ensure that it is complete and correct. If you have cited papers that have been retracted, please include the rationale for doing so in the manuscript text, or remove these references and replace them with relevant current references. Any changes to the reference list should be mentioned in the rebuttal letter that accompanies your revised manuscript. If you need to cite a retracted article, indicate the article’s retracted status in the References list and also include a citation and full reference for the retraction notice.

Response:We have carefully reviewed the entire reference list and confirmed that all references are complete, correct, and none have been retracted.

Additional Editor Comments :

Comment 1: Even though the manuscript studies parental anxiety, a little more literature reviews would be helpful, where the following studies can be used: Khoshlessan and Das, 2017; Tang et al. (2022)

Response: We thank the editor for this suggestion. We have incorporated the two recommended references into the opening paragraph of the Introduction: “In recent years, a growing body of research has examined education-related anxiety, including students’ academic anxiety and parental educational anxiety [1,2]. The latter has become a notable concern worldwide, particularly in East Asian contexts such as China [3].”

(See Line 43– 45 in the first paragraph of Section “1. Introduction”)

Comment 2: The use of hierarchical regression is reasonable; however, regression analysis for data containing high leverage points cane be discussed in the literature review (See Dey, et al. (2015) for more information).

Response: We greatly appreciate this methodological suggestion. We have carefully read and cited the reference you recommended (See Line 203 – 205 in the first paragraph of Section “2.5. Statistical analysis”): “Diagnostic statistics were examined in accordance with standard regression diagnostic procedures, with the aim of detecting potential influential observations (e.g., high leverage points) [55].” Accordingly, we showed the results of diagnostic statistics in the Results section (See Line 280– 283 in the first paragraph of Section “3.2.3. Predictors of PPEA”): “All VIFs were below 5, indicating no obvious multicollinearity. The Durbin-Watson statistic was 1.938, falling within the acceptable range of 1.5 to 2.5, thereby supporting independent error terms. The maximum Cook’s distance was 0.01, far below the critical cutoff of 1.0, indicating no influential outliers. These results verified that the assumptions for linear regression were sufficiently satisfied.” We believe this addition strengthens the methodological transparency of our regression analyses.

Response to Reviewer 1

Comment 1: Assessment of Technical Soundness: Based on the information provided, the study appears technically sound overall. The authors used separate samples to develop and validate the revised scale and provided evidence that the instrument is both reliable and consistent. The statistical analyses appear appropriate for addressing the study objectives, and the conclusions are well supported by the data and findings presented.

Comment 2: The statistical methods appear appropriate and rigorous for addressing the study objectives, including the use of exploratory and confirmatory factor analyses (EFA/CFA) for scale validation, reliability analyses, non-parametric tests, and hierarchical regression. The authors used separate samples to develop and validate the revised scale, reported strong reliability statistics (Cronbach's α = 0.922; ICC = 0.825), and employed an adequate sample size to examine factors associated with PPEA.

The authors demonstrate a thoughtful and appropriate interpretation of their findings throughout the manuscript. The discussion and conclusions are well aligned with the study design, statistical analyses, and the cross-sectional nature of the data. The discussion remains focused on associations supported by the findings, and the authors avoid overstating causal relationships. In particular, the use of the terms "risk factor" and "protective factor" is appropriate and reflects observed associations rather than causal claims, resulting in a balanced and scientifically sound interpretation of the results. The conclusions are generally consistent with the findings, including the associations between family conflict and higher PPEA and between resilience and lower PPEA.

Response: We sincerely thank you for your thorough and positive evaluation of our statistical approach. We are encouraged by your recognition that our interpretation remains well aligned with the cross-sectional design.

Comment 3: Availability of Data: The manuscript includes the revised PPEAS instrument, coding information, and detailed summary tables describing the statistical analyses and findings. However, the Data Availability Statement indicates that the data are available from the corresponding author upon request. To align with the journal's data availability requirements, the authors may consider providing access to the underlying de-identified dataset by depositing the data in a public repository or providing them as supporting information. Making the data publicly accessible would strengthen the transparency of the study.

Response: Thank you for your detailed suggestion regarding data transparency. In response to this comment, we have prepared the de-identified dataset and uploaded it as supplementary materials (S2_Dataset). Accordingly, we have revised our data availability statement in the manuscript to “Data analyzed in this study were available from the supplementary materials (Supplementary 2: dataset).”

(See Line 406)

Comment 4: Manuscript Presentation, Organization, Language, and Writing Quality: The manuscript is generally understandable and well organized. The clear structure of the manuscript, consisting of the Introduction, Materials and Methods, Results, Discussion, and Conclusion sections, makes it easy to follow and enhances readability.

However, the manuscript contains numerous grammatical errors and non-standard English expressions. Careful language editing is recommended to improve clarity, readability, and adherence to standard academic English. Examples include:

A) Line 28: "for confirmatory factor analysis (EFA) and reliability testing" appears to contain a typographical error. "EFA" should be revised to "CFA" (Confirmatory Factor Analysis).

B) Line 23, 24: "This study aimed to revise the Parental Educational Anxiety Scale from parents' perspective to children's angle." Consider revising to: "This study aimed to adapt the Parental Educational Anxiety Scale from the parents' perspective to the children's perspective."

C) Line 29: "646 participants completed revised PPEAS..." Consider revising to: "646 participants completed the revised PPEAS..."

D) Line 39: "providing a reliable tool for measuring perceived educational anxiety from children's perspective." Consider revising to: "providing a reliable tool for measuring perceived parental educational anxiety from the children's perspective."

E) Line 81: "Moreover, PPEA may be influenced by some factors." Consider revising to: "Moreover, PPEA may be influenced by several factors."

F) Line 211: "The result showed suitable for factor analysis" Consider revising to: "The results showed that the data were suitable for factor analysis."

G) Line 232: Please revise the phrase "less paternal emotional warmth" to "lower parental emotional warmth" for clarity, as both paternal and maternal emotional warmth are reported in this section.

H) Line 277: Please revise "indicating these three factors are not isolate but interrelated." to "indicating these three factors are not isolated but interrelated."

I) Line 315, 316: The sentence contains a grammatical error. Please revise "which not only increase their own educational anxiety but also pressure on their children." to "which not only increase their own educational anxiety but also place pressure on their children."

J) Line 340 - 342: Please provide an appropriate citation to support the statement: "Moreover, prior research indicates that highly resilient individuals possess a great capacity for cognitive reappraisal, which may enable them to reinterpret their parents' educational anxiety as a form of involved concern rather than a threatening signal, thereby contributing to lower PPEA."

Overall, this manuscript addresses an important topic and has the potential to make a meaningful contribution to the literature. Addressing the concerns above would improve the clarity, transparency, and overall quality of the manuscript.

Response: We sincerely thank you for carefully identifying these specific language issues, and we have corrected all the highlighted errors point-by-point as follows:

A) The original phrase “for confirmatory factor analysis (EFA) and reliability testing” has been revised to “for confirmatory factor analysis (CFA) and reliability testing.”(See Line 28 in the Abstract

B) The original sentence “This study aimed to revise the Parental Educational Anxiety Scale from parents’ perspective to children’s angle.” has been revised to “This study aimed to revise the Parental Educational Anxiety Scale from a parental perspective to a child-perceived perspective.”(See Line 22 – 23 in the Abstract)

C) The original phrase “646 participants completed revised PPEAS...” has been revised to “646 participants completed the revised PPEAS.” (See Line 28 in the Abstract)

D) The original phrase “providing a reliable tool for measuring perceived educational anxiety from children's perspective” has been revised to “providing a reliable instrument for measuring perceived educational anxiety from the children’s perspective”.(See Line 37 – 38 in the Abstract)

E) The original sentence “Moreover, PPEA may be influenced by some factors.” has been revised to “Moreover, PPEA may be influenced by several factors.” (See Line 80 in the Introduction)

F) The original phrase “The result showed suitable for factor analysis” has been revised to “The results confirmed the suitability of the data for factor analysis.” (See Line 223 in the Results)

G) The original phrase “less paternal emotional warmth” has been revised to “significantly and negatively correlated with parental emotional warmth”.(See Line 248-249 in Section “3.1.5. Criterion-related validity”)

H) The original phrase “indicating these three factors are not isolate but interrelated” has been revised to “indicating that these three factors are not isolated but interrelated”.(See Line 303 – 304 in the first paragraph of Section “4.1. The revised scale has good reliability and validity with a reasonable structure”)

I) The original phrase “which not only increase their own educational anxiety but also pressure on their children.” has been revised to “as such expectations not only elevate their own educational anxiety but also place pressure on their children.” (See Line 340-341 in the second paragraph of Section “4.2. Chinese college freshmen reported a relatively low PPEA, with higher scores in males”)

J) Thank you for pointing out this missing reference. We have added a citation to support the statement regarding cognitive reappraisal in resilient individuals: “As indicated by existing research, highly resilient individuals possess a great capacity for cognitive reappraisal [31], which may enable them to reinterpret their parents’ educational anxiety as a form of involved concern rather than a threatening signal. Thus,lower PPEA may be achieved.”(See Line 364-367 in the first paragraph of Section “4.3. Influences of family conflict, family cohesion and children’s resilience on PPEA”)

Beyond addressing each specific issue you raised above, we have had the entire manuscript professionally copyedited to correct the grammatical errors and ensure language clarity and readability. The editing certificate has been listed here.

Response to Reviewer 2

Major Comments

Comment 1: The scale revision process requires stronger justification and evidence of content validity.

The central contribution of the manuscript is the revision of the Parental Educational Anxiety Scale (PEAS) into the Perceived Parental Educational Anxiety Scale (PPEAS). However, the revision process appears to consist prim

Attachment Submitted filename: Respons to reviews PPEA R1.docx

# Peer review: Decision Letter 1

**DOI:** 10.1371/journal.pone.0358097.r003

**Authors:** Kumer Das

26 Aug 2026

Revision of the perceived parental-educational-anxiety scale and its application among Chinese college freshmen

PONE-D-26-23832R1

Dear Dr. Dai,

We’re pleased to inform you that your manuscript has been judged scientifically suitable for publication and will be formally accepted for publication once it meets all outstanding technical requirements.

Within one week, you’ll receive an e-mail detailing the required amendments. When these have been addressed, you’ll receive a formal acceptance letter and your manuscript will be scheduled for publication.

An invoice will be generated when your article is formally accepted. Please note, if your institution has a publishing partnership with PLOS and your article meets the relevant criteria, all or part of your publication costs will be covered. Please make sure your user information is up-to-date by logging into Editorial Manager at Editorial Manager® and clicking the ‘Update My Information' link at the top of the page. For questions related to billing, please contact billing support.

If your institution or institutions have a press office, please notify them about your upcoming paper to help maximize its impact. If they’ll be preparing press materials, please inform our press team as soon as possible -- no later than 48 hours after receiving the formal acceptance. Your manuscript will remain under strict press embargo until 2 pm Eastern Time on the date of publication. For more information, please contact onepress@plos.org.

Kind regards,

Kumer Das

Academic Editor

PLOS One

Additional Editor Comments (optional):

Reviewers' comments:

Reviewer's Responses to Questions

**Comments to the Author**

Reviewer #1: All comments have been addressed

Reviewer #2: (No Response)

**********

2. Is the manuscript technically sound, and do the data support the conclusions?

Reviewer #1: (No Response)

Reviewer #2: Yes

**********

3. Has the statistical analysis been performed appropriately and rigorously?

Reviewer #1: (No Response)

Reviewer #2: Yes

**********

4. Have the authors made all data underlying the findings in their manuscript fully available?

The PLOS Data policy

Reviewer #1: (No Response)

Reviewer #2: Yes

**********

5. Is the manuscript presented in an intelligible fashion and written in standard English?

Reviewer #1: (No Response)

Reviewer #2: Yes

**********

Reviewer #1: The authors have adequately addressed the comments and concerns I raised in the previous round of review. I am satisfied with the revisions made and believe that the manuscript is now suitable for publication.

Reviewer #2: Thank you for the detailed response to the previous review and for the substantial revisions made to the manuscript. I appreciate the your careful attention to the concerns raised during the initial review. In particular, the additional information regarding the scale adaptation and content-validity procedures, regression diagnostics, common method bias, sampling limitations, retrospective recall, measurement invariance, data availability, and language editing has substantially strengthened the manuscript. I consider my primary methodological and statistical concerns from the initial review to have been adequately addressed.

I now consider the manuscript technically sound overall, the statistical analyses appropriate and sufficiently justified, the underlying data adequately available through the supporting information, and the manuscript substantially improved in clarity and presentation. I believe only a few minor points require attention before the manuscript is suitable for publication.

1. Abstract – EFA/CFA reporting. My previous review requested that the Abstract clearly distinguish the EFA findings from the CFA model-fit results. Although the authors corrected “EFA” to “CFA” when describing the analysis conducted with Sample 2, the Results portion of the Abstract still states that “EFA identified a three-factor structure consisting of 16 items with adequate model fit (χ²/df = 2.691, RMSEA = 0.077, TLI = 0.919, CFI = 0.932).” These model-fit indices correspond to the CFA rather than the EFA. Please separate the EFA finding from the CFA model-fit findings so that the Abstract accurately represents the analyses performed.

2. Interpretation of the proposed perception gap. I appreciate the your addition to the Limitations acknowledging that parental educational anxiety was not directly assessed and that the criterion-related validity evidence remains indirect. Consistent with this acknowledgment, I recommend slightly qualifying the statement in the Conclusion that the findings suggest a “meaningful perception gap.” Because the comparison is based on findings from different studies rather than paired parent-child observations, the present study can identify a cross-study difference but cannot directly establish a parent-child perception gap. This could instead be presented as an observation warranting future investigation using the parent-child dyad design already appropriately recommended in the Limitations.

3. Consistency of non-causal interpretation. You have substantially addressed my previous concern regarding causal terminology and appropriately replaced terms such as “risk factor” and “protective factor” with associative language. I recommend one final consistency check for statements suggesting that reducing family conflict or enhancing resilience may lower or mitigate PPEA. Given the cross-sectional design, these implications would be more appropriately framed as potential directions for future longitudinal or intervention research rather than as effects demonstrated by the present study.

Overall, the you have responded thoroughly and constructively to the initial review, and the manuscript has improved substantially. My previous concerns regarding technical soundness, statistical analysis, data availability, and language quality have been satisfactorily addressed. The remaining points are limited in scope and primarily concern precision and consistency in reporting and interpretation rather than fundamental methodological deficiencies. I therefore recommend Minor Revision. Following these limited corrections, I believe the manuscript would be suitable for publication in PLOS ONE.

**********

what does this mean?). If published, this will include your full peer review and any attached files.

If you choose “no”, your identity will remain anonymous but your review may still be made public.

**Do you want your identity to be public for this peer review?** For information about this choice, including consent withdrawal, please see our Privacy Policy

Reviewer #1: No

Reviewer #2: No

**********

# Editor report: Acceptance letter

**DOI:** 10.1371/journal.pone.0358097.r004

**Authors:** Kumer Das

PONE-D-26-23832R1

PLOS One

Dear Dr. Dai,

I'm pleased to inform you that your manuscript has been deemed suitable for publication in PLOS One. Congratulations! Your manuscript is now being handed over to our production team.

At this stage, our production department will prepare your paper for publication. This includes ensuring the following:

* All references, tables, and figures are properly cited

* All relevant supporting information is included in the manuscript submission,

* There are no issues that prevent the paper from being properly typeset

You will receive further instructions from the production team, including instructions on how to review your proof when it is ready. Please keep in mind that we are working through a large volume of accepted articles, so please give us a few days to review your paper and let you know the next and final steps.

Lastly, if your institution or institutions have a press office, please let them know about your upcoming paper now to help maximize its impact. If they'll be preparing press materials, please inform our press team within the next 48 hours. Your manuscript will remain under strict press embargo until 2 pm Eastern Time on the date of publication. For more information, please contact onepress@plos.org.

You will receive an invoice from PLOS for your publication fee after your manuscript has reached the completed accept phase. If you receive an email requesting payment before acceptance or for any other service, this may be a phishing scheme. Learn how to identify phishing emails and protect your accounts at https://explore.plos.org/phishing.

If we can help with anything else, please email us at customercare@plos.org.

Thank you for submitting your work to PLOS One and supporting open access.

Kind regards,

PLOS One Editorial Office Staff

on behalf of

Dr. Kumer Das

Academic Editor

PLOS One
